# Comparison of Fatty Acid and Gene Profiles in Skeletal Muscle in Normal and Obese C57BL/6J Mice before and after Blunt Muscle Injury

**DOI:** 10.3389/fphys.2018.00019

**Published:** 2018-01-30

**Authors:** Jens-Uwe Werner, Klaus Tödter, Pengfei Xu, Lydia Lockhart, Markus Jähnert, Pascal Gottmann, Annette Schürmann, Ludger Scheja, Martin Wabitsch, Uwe Knippschild

**Affiliations:** ^1^Department of General and Visceral Surgery, Ulm University Hospital, Ulm, Germany; ^2^Department of Biochemistry and Molecular Cell Biology, University Medical Center Hamburg-Eppendorf, Hamburg, Germany; ^3^Department of Experimental Diabetology, German Institute of Human Nutrition, Potsdam, Germany; ^4^Division of Pediatric Endocrinology and Diabetes, University Hospital for Pediatrics and Adolescent Medicine, Ulm, Germany

**Keywords:** obesity, injury, C57BL/6J, skeletal muscle, fatty acid, *Alox5ap*, *Apobec1*

## Abstract

Injury and obesity are two major health burdens affecting millions of people worldwide. Obesity is recognized as a state of chronic inflammation accompanied by various co-morbidities like T2D or cardiovascular diseases. There is increasing evidence that obesity impairs muscle regeneration, which is mainly due to chronic inflammation and to excessive accumulation of lipids in adipose and non-adipose tissue. To compare fatty acid profiles and changes in gene expression at different time points after muscle injury, we used an established drop tower-based model with a defined force input to damage the *extensor iliotibialis anticus* on the left hind limb of female C57BL/6J mice of normal weight and obese mice. Although most changes in fatty acid content in muscle tissue are diet related, levels of eicosaenoic (normal weight) and DHG-linolenic acid (obese) in the phospholipid and docosahexaenoic acid (normal weight) in the triglyceride fraction are altered after injury. Furthermore, changes in gene transcription were detected in 3829 genes in muscles of normal weight mice, whereas only 287 genes were altered in muscles of obese mice after trauma. Alterations were found within several pathways, among them notch-signaling, insulin-signaling, sonic hedgehog-signaling, apoptosis related pathways, fat metabolism related cholesterol homeostasis, fatty acid biosynthetic process, fatty acid elongation, and acyl-CoA metabolic process. We could show that genes involved in fat metabolism are affected 3 days after trauma induction mostly in normal weight but not in obese mice. The strongest effects were observed in normal weight mice for *Alox5ap*, the activating protein for leukotriene synthesis, and *Apobec1*, an enzyme substantial for LDL synthesis. In summary, we show that obesity changes the fat content of skeletal muscle and generally shows a negative impact upon blunt muscle injury on various cellular processes, among them fatty acid related metabolism, notch-, insulin-, sonic hedgehog-signaling, and apoptosis.

## Introduction

Approximately 1.9 billion adults worldwide were considered to be overweight or obese in 2014, while affecting 41 million children in parallel mostly due to a sedentary western life-style and over-nutrition (WHO, 2016). Obesity is characterized by an increase in size and number of adipocytes in white adipose tissue (WAT) and an impaired adipocyte-dependent function (Adamczak and Wiecek, 2013; Richard and Stephens, 2014; Sinha et al., 2017). WAT is a complex endocrine organ secreting a wide range of hormones and other factors thereby influencing physiological processes in numerous other tissues (Adamczak and Wiecek, 2013). Obesity is frequently associated with several co-morbidities, among them type 2 diabetes, cardiovascular diseases, and musculoskeletal disorders (WHO, 2016; Sinha et al., 2017). Obesity usually leads to a chronic state of inflammation resulting in an increased infiltration and impaired polarization of macrophages, which in turn results in elevated levels of inflammatory cytokines and reactive oxygen species within the WAT (Akhmedov and Berdeaux, 2013; D'Souza et al., 2015; WHO, 2016). Furthermore, circulating free fatty acids (FA) are elevated in obese individuals leading to an increase in intramyocellular and extramyocellular lipids in muscle tissue (Boden and Shulman, 2002; Sinha et al., 2002; Schrauwen-Hinderling et al., 2003; Vogt et al., 2003). This so called ectopic lipid accumulation gives rise to lipotoxicity negatively influencing cell signaling and metabolism (Hulver et al., 2003; Adams et al., 2004; Guilherme et al., 2008; Muoio and Neufer, 2012; Akhmedov and Berdeaux, 2013).

Skeletal muscle comprises around 40% of the human body while being exposed to everyday activity, thus being prone to injuries. This requires a continuous repair of damaged fibrils and regrowth of new myofibers (Yin et al., 2013; Ceafalan et al., 2014; Frontera and Ochala, 2015). Muscle regeneration upon injury is a highly complex and synchronized process that involves several intertwined phases: Trauma, a disruption of muscle tissue, triggers the multistep regeneration process, which consists of an inflammatory response at the beginning that leads to the recruitment of immune cells. Necrotic myofibers are removed by neutrophils and M1-type macrophages (MΦ), while exerting a pro-inflammatory program to activate satellite cells and to promote their proliferation. Thereafter, M2-type MΦ secrete anti-inflammatory mediators that lead to differentiation of satellite cells into myoblasts enabling their fusion and regrowth to fully functional muscle fibers within a few weeks (Akhmedov and Berdeaux, 2013; Yin et al., 2013; Ceafalan et al., 2014; Forbes and Rosenthal, 2014; Rigamonti et al., 2014; D'Souza et al., 2015; Hardy et al., 2016). MΦ, although described in a simplified way, are key players in muscle regeneration. They regulate activation, proliferation and differentiation of satellite cells by releasing specific signaling molecules at each phase of the regeneration process (Smith et al., 2008; Akhmedov and Berdeaux, 2013; Pillon et al., 2013; Forbes and Rosenthal, 2014; Rigamonti et al., 2014). Muscle regeneration is susceptible to lipid burden in obese individuals (Tamilarasan et al., 2012; Akhmedov and Berdeaux, 2013). Hence, elevated levels of lipid metabolites, cytokines and altered MΦ functions impair satellite cell activation and functions in obese mice thereby affecting regenerative capacity (Adamczak and Wiecek, 2013; Akhmedov and Berdeaux, 2013; D'Souza et al., 2015).

Obesity is known to be associated with impaired muscle regeneration (Akhmedov and Berdeaux, 2013; D'Souza et al., 2015; Sinha et al., 2017). However, only few studies have combined diet-induced obesity by high fat diet (HFD) with a blunt injury animal model. Hardy et al. (2016) evaluated the most common injury models in mice, namely freezing, barium chloride, notexin and cardiotoxin, which apply either physical, chemical or toxic strain on the muscle and showed that different kinds of injuries may lead to different responses with regard to regeneration. In this work, we used an improved drop tower-based model with a defined force input to damage the muscle *extensor iliotibialis anticus* of normal weight and obese mice either fed a control or high fat diet. In order to investigate the influence of obesity-related factors on skeletal muscle regeneration after induction of a blunt trauma, we assessed different nutritional conditions and their influence on FA content and FA-metabolism related genes at different stages of the regeneration processes. Our results revealed that levels of eicosaenoic (normal weight) and DHG-linolenic acid (obese) in the phospholipid and docosahexaenoic acid (normal weight) in the triglyceride fraction are altered after injury, and that injury directly influences FA-metabolism by altering *Alox5ap* and *Apobec1* expression.

Transcriptome analysis revealed a total of 3829 differently expressed genes (DEG) in normal weight mice within the whole time course after injury, whereas only 272 DEG were found in obese animals. Changes were detected within several pathways, among them fat metabolism related cholesterol homeostasis, fatty acid biosynthetic process, fatty acid elongation and acyl-CoA metabolic process with high confidence. Additionally, changes in the expression levels of several genes involved in apoptosis, notch-, insulin-, and sonic hedgehog-signaling were observed in muscle tissue of obese and normal weight mice. In summary, our results highlight the negative impact of obesity on several cellular processes in skeletal muscle after injury.

## Materials and methods

### Animal housing and breeding

Sixteen-week-old male and female C57BL/6J mice were purchased from the in-house breeding facility of Ulm University, and kept in a pathogen free open cage facility in a 12 h light/dark cycle at 22.5 ± 1°C with access to food and water *ad libitum*. Animals were used for breeding purposes until exhausted by law. Parental animals received a normal (ND; 10% kcal fat; D12450J) or high fat diet (HFD; 60% kcal fat; D12492, purchased from Research Diets Inc. by their European distributor Brogaarden®, Gentofte, Denmark.), started 1 week prior to breeding to influence prenatal development of litter. Litters received the corresponding parent diet. Only female mice were used for trauma induction. All animal experiments were approved by state and local authorities (license: 1183) and were carried out in accordance with ARRIVE guidelines and local regulations.

### Traumatic muscle injury

Prior to application of injury 16 ± 1 week old normal weight and obese C57BL/6J mice were anesthetized with 2.5 vol% sevoflurane (Sevorane^TM^ Abbott, Wiesbaden, Germany) mixed with 97.5 vol% oxygen. Analgesic treatment involved s.c. injection of buprenorphine (Temgesic® Reckitt Benckiser, Berkshire, Great-Britain [0.03 mg/kg]). The area around the muscle to be hit was shaved and marked to increase reproducibility. Control animals did not receive any injury. Induction of injury to *extensor iliotibialis anticus* muscle of the left hind leg was then carried out by a mechanical drop tower device, similarly as recently described for the facial nerve (Wanner et al., 2017). Briefly, the left hind limb was positioned under the drop tower, and fixed onto a scaffold. A wedge with a flat surface was placed onto *extensor iliotibialis anticus*, allowing a weight of 40 g being dropped to generate an indirect but controlled force input. Effective drop height was 104 cm. A removable spacer (3 mm) on top of a crossbar to limit the total depth of penetration was used to avoid bone fracture. Female C57BL/6J litter animals were randomly grouped either into the “blunt muscle injury” (*n* = 12 per time point) or the “control” (*n* = 6 per time point) group and sacrificed by CO_2_ inhalation at different time points of the regeneration process (1, 6, 24 h, 3, 8, and 21 d). Tissue from *extensor iliotibialis anticus* of normal weight and obese groups was isolated, snap frozen in liquid nitrogen and stored at −80°C until further use.

### Hematoxylin and eosin (HE) staining

Muscle tissue of the *extensor iliotibialis anticus* from untreated or injured normal weight and obese mice were collected at different time points. The specimens were embedded in paraffin, and processed for 5 μm thick cross-sections with circular layer and myenteric ganglia cut longitudinally. After deparaffinization specimen were stained for 3 min in hematoxylin (Sigma-Aldrich, Munich, Germany). Thereafter specimen were washed 2 times with H_2_O followed by incubation with 1% acidic alcohol for 3 s and re-staining with running tap water for 10 min. Then, cytoplasm of specimens was stained with eosin (Sigma-Aldrich, Munich, Germany) for 10 s. Dehydration of sections was performed two times by incubation in 96% EtOH for 5 min, followed by two times incubation in 100% EtOH for 5 min. Thereafter, specimens were incubated in Roti®-Histol for 10 min. Sections were mounted using Entellan®.

### Lipid extraction and gas chromatography

Muscle tissue was extracted by adding 10 μl chloroform-methanol (2:1 vol.) per mg tissue and homogenized using a bead mill (TissueLyzer, Qiagen, Hilden, Germany). After centrifugation, aliquots were spotted on silica thin layer chromatography plates (Macherey and Nagel, Düren, Germany) and developed with hexane-diethyl ether-acetic acid (80:20:1.5 vol.). PL and TG fractions were visualized with primuline, scratched off and transferred into centrifuge vials. Fatty acid methyl ester were prepared as described by Lepage and Roy (Lepage and Roy, 1986) except that toluene was used instead of benzene. Briefly, 1 ml methanol-toluene (4:1), 100 μl heptadecanoic acid (200 μg/ml in methanol-toluene 4:1), 25 μl butylated hydroxytoluene (0.1 M in methanol) and 100 μl acetyl chloride were added to the scratched silica samples and heated to 100°C for 1 h. After addition of 3 ml 6% sodium carbonate and centrifugation 50–100 μl of the upper layer were transferred into gas chromatography vials with inserts. Gas chromatography analyses were performed using a HP 5890 gas chromatograph equipped with FID and a DB225 column (both from Agilent, Waldbronn, Germany). Peak identification and quantification was performed by comparing retention times and peak areas, respectively, to standard chromatograms. All calculations are based on fatty acid methyl esters values.

### RNA isolation and microarray analysis

Muscle tissue from female normal weight and obese animals (*n* = 3) as well as from control and trauma mice was used from each time point (1, 6, 24 h, 3 and 8 d). Total RNA was isolated using the RNeasy Mini Kit (Qiagen, Hilden, Germany) following the manufacturer's protocol. Microarray analysis was carried out using the Affymetrix (Thermo Fisher Scientific, Dreieich, Germany) system in our in-house Genomics core facility. RNA was analyzed on integrity, contamination with genomic DNA and feasibility using the Agilent 2100 Bioanalyzer System. One hundred nanogram RNA was used as described in the GeneChip® WT PLUS Reagent Kit from Affymetrix, while using Mouse Gene 1.0 ST Array GeneChip (Thermo Fisher Scientific, Dreieich, Germany). Arrays were analyzed with Affymetrix GeneChip Scanner 3000 and first evaluated using Affymetrix Expression Console™. A method to evaluate the CEL files was designed with RStudio in R. Briefly, control versus injured animals were RMA-normalized for time and diet, respectively. *P*-values were determined using the pval-function. Up-/down-regulation was assessed without considering significance or expression levels in order to generate an unbiased time lapse. Basal level of controls by this method equals ±1. Significance of *p* ≤ 0.05 was set to determine significant changes and to find potential candidates for further validation in a RT-qPCR approach. This led to the identification of most prominent genes expressed over the whole time course up to 8 days with focus on fatty acid-related genes. The complete data set is accessible at Gene Expression Omnibus (GSE103726).

### Real-time qPCR

One hundred nanogram of total RNA was transcribed into cDNA using the AffinityScript cDNA Synthesis Kit (Agilent Technologies, Santa Clara, USA) according to manufacturer's instructions. *Ppia* (*Mm_Ppia_1_SG QT00247709*) was used as reference gene according to Gong et al. (2016). Marker genes were *Alox5ap* (*Mm_Alox5ap_1_SG QT01050574*) and *Apobec1* (*Mm_Apobec1_1_SG QT00109844*). Reference genes, markers and SYBR® Green (QantiTect® SYBR® Green PCR Kit) were purchased from Qiagen (Hilden, Germany). For data evaluation, each well was related to the average of the housekeeping gene, then averaged again in order to get a value per mouse. Mice were then grouped appropriate to time of treatment (control/injury) and diet. Each injured subgroup was normalized on their corresponding controls to determine x-fold values.

### Statistical evaluation

Statistical analysis was carried out by two-sided homoscedastic *t*-test, for weight distribution and comparisons of fatty acid profiles in phospholipid and triglyceride fractions. Significance levels for differences in fatty acid ratios were calculated using Fisher's exact test in combination with odds ratio for each variable (SFA, MUFA and PUFA). Calculation was performed by R, using the “questionr” package (see Supplement 1.1). Microarray evaluation used the pval-function in R to determine significance. Comparison of qPCR results was performed with two factorial analysis of variance (ANOVA) with repeated measurements (*n* = 3).

### Software

All pictures were generated using GraphPad Prism version 5 for Windows, GraphPad Software, San Diego California USA, www.graphpad.com.

CEL files were analyzed with RStudio (RStudio Team, 2016) (version 0.99.903), R (R Development Core Team, 2016) (version 3.2.5) and various R-packages (e.g., “affy,” “pvclust,” “gplots,” and “FactoMineR”). For a complete list of the packages including version numbers and citations, please refer to Supplement 1.2.

Pathway analysis was carried out with ClueGO (Bindea et al., 2009) v2.3.3, a plug-in for Cytoscape (Lotia et al., 2013).

## Results

### General characteristics

**Figure 1:**
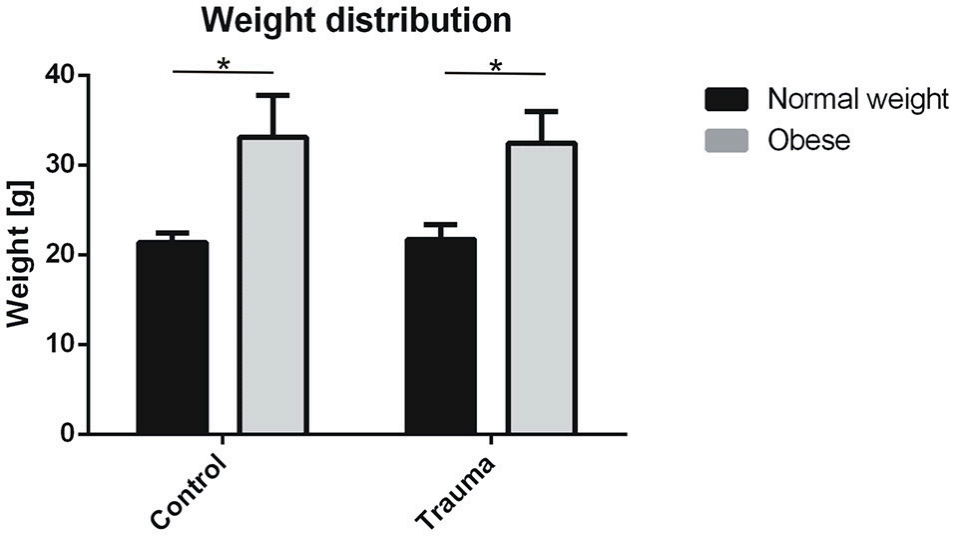
Body weight of animals and significant differences of our cohort. Values are given with mean ±standard deviation; *n* = 18 per group. Statistical analysis by two-sided homoscedastic *t*-test. *Indicates *p* ≤ 0.01.

In order to assess obesity and trauma induced alterations in fatty acid content in skeletal muscle, an established muscle trauma model was used in which a defined drop tower device induced a blunt injury to the left hind leg of female 16 ± 1 weeks old normal weight and obese C57BL/6J littermates. General characteristics for diet, strain, sex, treatment, sample size, age, and body weight are depicted in Table 1. Since nutrition during pregnancy and early lifetime already has a great impact on muscle physiology and regeneration capacity (Woo et al., 2011; Hemalatha, 2013), already 1 week prior to breeding, parent animals as well as their littermates received either a ND (10% kcal) or HFD (60% kcal fat). Whereas diet had a significant influence on total body weight (*p* ≤ 0.01; Figure 1), blunt muscle injury did not result in any significant changes in the body weight of normal weight and obese mice post-injury (Table 2).

**Table 1:** Characteristics for animals used for induction of a blunt muscle trauma.

	**Normal weight**		**Obese**	
Diet	D12450J		D12492	
	Normal (ND)		High fat (HFD)	
	10% kcal fat		60% kcal fat	
Strain	C57BL/6J			
**Sex**	**Female**			
**Treatment**	**Control**	**Trauma**	**Control**	**Trauma**
n =	18	18	18	18
Age [week]	16.3 ± 0.69	16.5 ± 0.52	16.3 ± 0.73	16.4 ± 0.69
Body weight [g]	21.4 ± 1.02	21.7 ± 1.67	33.1 ± 4.67	32.4 ± 3.54

*Age and body weight are depicted as mean ± standard deviation*.

**Table 2:** Effect of blunt muscle injury on the weight of normal weight and obese animals.

	**Normal**				**Obese**			
	**Before**		**After**		**Before**		**After**	
**CONTROL**								
1 h	21.4	±1.74	n.m.		31.2	±5.48	n.m.	
6 h	21.3	±1.33	n.m.		32.6	±1.91	n.m.	
24 h	20.0	±1.15	19.8	±0.70	25.4	±4.56	24.4	±4.08
3 d	19.5	±0.57	20.1	±0.75	28.0	±5.84	27.9	±5.76
8 d	20.7	±1.19	21.1	±1.95	31.2	±0.51	31.7	±0.32
**TRAUMA**								
1 h	22.4	±0.78	n.m.		28.3	±4.57	n.m.	
6 h	22.5	±1.39	n.m.		34.9	±5.00	n.m.	
24 h	21.2	±0.36	20.5	±0.46	26.3	±4.31	26.4	±4.18
3 d	20.9	±1.42	20.7	±1.46	29.3	±0.40	27.6	±2.01
8 d	22.1	±2.82	21.6	±1.86	29.6	±2.75	29.3	±2.56

*All animals were weighed before and at different time points after muscle injury. Weight loss was only noted after trauma with slight decreases for both, normal weight and obese animals. Values are depicted as average in grams ±standard deviation per group; n.m., not measured*.

**Figure 2:**
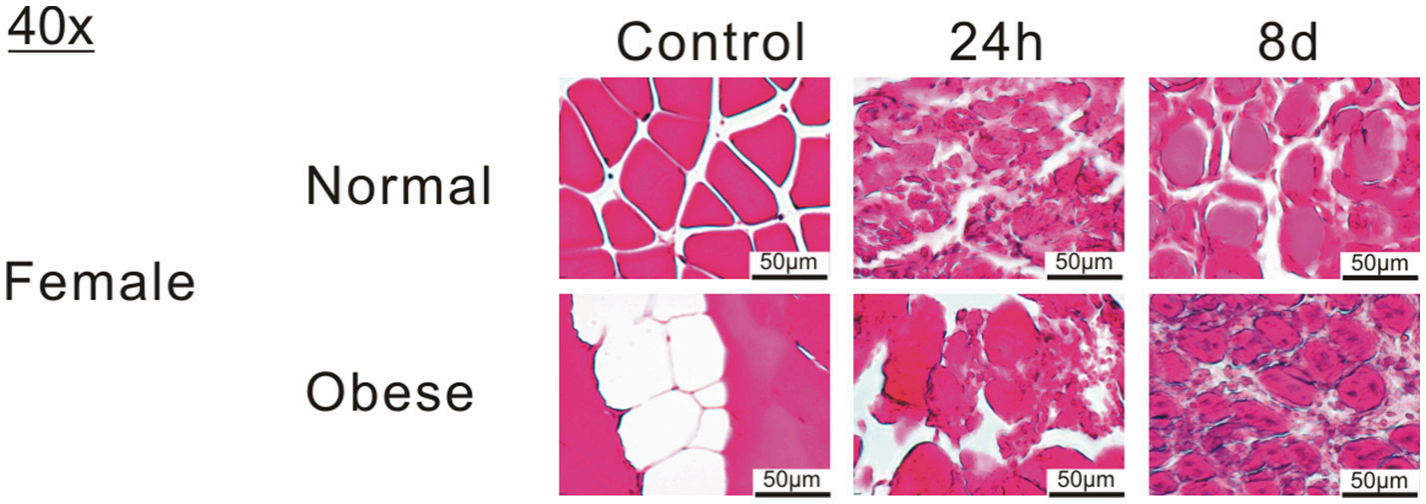
Hematoxylin-eosin (HE) staining of muscle tissue sections from female normal weight and obese mice for control, 24 h and 8 d after blunt injury induction. Muscle damage and myofiber regeneration are visible. Pictures were taken with Olympus IX81 using Xcellence v.1.2. Scale = 50 μm at 40x magnification.

Although normal weight and obese mice differ significantly in weight, no statistically significant differences in the impact forces generated by the drop-tower device were detected (own unpublished data). Therefore, the degree of muscle damage was similar in both animal groups and led to formation of hematoma and edema in the injured muscle tissue of normal weight and obese mice peaking 24 h post-injury. In regard to morphology, regeneration has been much more advanced in normal weight mice than in obese mice 8 days post-injury (Figure 2 and Table 3).

**Table 3:** Blunt muscle injury to *extensor iliotibialis anticus* results in similar muscle damage but to an increased fibrosis formation in obese mice compared to normal weight mice.

	**Normal weight mice**	**Obese mice**
Interstitial fluid, edema and hemorrhage exudate	Formation of interstitial fluid, edema and hemorrhage exudate already 1 h after injury detectable	Interstitial fluid, edema and hemorrhage exudate formation as in normal weight animals
Morphology	Disruption of tissue, peak inflammation 24 h after injury. Regeneration was completed after 21 day	Disruption of tissue, peak inflammation 2 days delayed. Muscle regeneration still incomplete after 21 days

### Comparison of fatty acid ratios in normal weight and obese animals

Since elevated free fatty acids (FA) and lipid accumulation in obese individuals lead to lipotoxicity negatively influencing various cellular processes including regeneration and metabolism (Hulver et al., 2003; Adams et al., 2004; Guilherme et al., 2008; Muoio and Neufer, 2012; Akhmedov and Berdeaux, 2013), we focused on the fatty acid ratios in normal weight and obese mice before and at different times after blunt injury induction. Evaluation of the influence of time on muscle tissue FA composition after blunt muscle injury in normal weight and obese mice at different time points (1, 6, 24 h, 3, 8, and 21 d post-injury) revealed no effect of time on fatty acid composition, thus allowing to combine samples (see Supplements 2.2, 2.3, 2.5, 2.6). In order to determine the percentage estimate of saturated (SFA), monounsaturated (MUFA) and polyunsaturated (PUFA) fatty acid ratios in muscle in all groups, both, the phospholipid and triglyceride fractions were cumulated, respectively.

**Figure 3:**
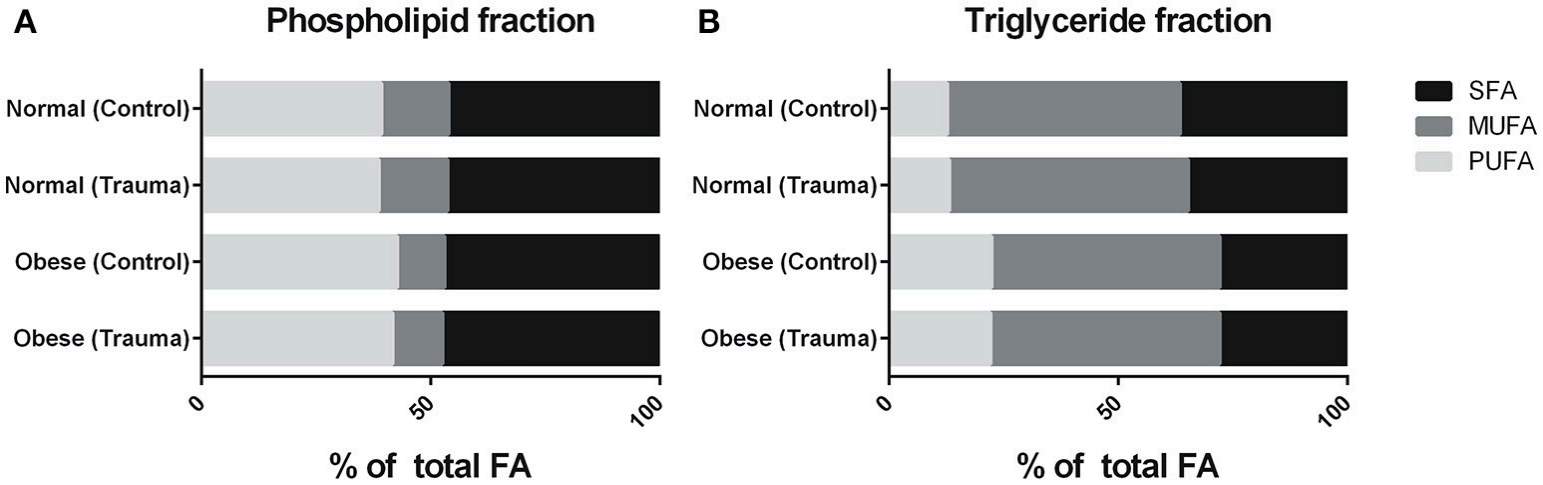
**(A)** Ratios grouped into saturated (SFA), monounsaturated (MUFA) and polyunsaturated (PUFA) fatty acids for phospholipid and triglyceride fractions respectively. Injury influenced MUFA in both groups and PUFA notably in obese mice in phospholipid fraction. SFA was higher in normal weight controls. Phospholipid SFAs did not change, while MUFAs were higher and PUFAs lower in normal weight mice **(B)**. Triglyceride SFAs were higher and PUFAs were lower in normal weight mice. MUFA content did not change. Trauma includes 1, 6, 24 h, 3, 8, and 21 d. NC, Normal (Control); NT, Normal (Trauma); OC, Obese (Control); and OT, Obese (Trauma).

The phospholipid fraction (Figure 3A) showed no change in SFA content following diet or injury (NC 46.16, NT 46.30, OC 46.90, and OT 47.32%). However, MUFA content was slightly higher in muscle tissue from trauma than in muscle tissue of control animals, while normal weight mice showed a higher content compared to obese mice (NC 14.62, NT 15.02, OC 10.35, and OT 10.94%). Conversely, normal weight animals showed less PUFA in muscle tissue, while trauma results in a slight decrease in PUFA content (NC 39.17, NT 38.57, OC 42.59, and OT 41.59%).

In the triglyceride fraction (Figure 3B), SFA content was higher in normal weight compared to obese animals. Control animals in this group were higher in SFA content than in animals of the trauma group (NC 36.73 vs. NT 34.93%), while obese muscle tissue showed no distinct change (OC 27.96 vs. OT 27.92%). MUFA content was similar in all groups (NC 50.81, NT 52.16, OC 49.78, and OT 50.05%). PUFA showed a strong decrease in muscle tissue from normal weight animals when compared to obese animals (NC 12.63% and NT 13.00% vs. OC 22.26% and OT 22.04%).

Cumulative values were used for Fisher's exact test and odds ratio to investigate significance and tendencies. In this regard, no statistical significance was seen when combining all increments. Furthermore, cumulated FA content of skeletal muscle is independent of injury, while diet is able to exhibit slightly higher odds of increased FA content (see Supplement 1.1).

### Fatty acid composition of phospholipid fraction

Muscle tissue of normal weight and obese mice collected at all-time points (1h up to 21d) was used for isolation of fatty acids to determine changes in lipid metabolites. The phospholipid fraction was isolated by thin-layer chromatography and analyzed by gas chromatography (Table 4). Myristoleic, stearidonic, DHG-linolenic, eicosatetraenoic, and eicosapentaenoic acid could not be detected. However, palmitic, stearic, arachidonic, and docosahexaenoic acid reached over 10% of total fatty acid fraction. Muscle tissue from trauma animals showed a significant influence on eicosenoic (ND, CvT) and DHG-linolenic acid (HFD, CvT) with *p* ≤ 0.05, while the other FAs remained unaffected (see Supplement 2.1). In contrast, diet revealed a strong effect on almost all fatty acids when comparing normal weight vs. obese tissue for control and trauma, respectively.

**Table 4:** FA-composition of phospholipid fraction in muscle tissue.

	**Phospholipid fraction**							
	**Normal**				**Obese**			
	**Control**		**Trauma**		**Control**		**Trauma**	
Myristic (14:0)	1.106	±0.045	1.160	±0.345	0.779	±0.081	0.771	±0.054
Myristoleic (14:1)	n.d.		n.d.		n.d.		n.d.
Palmitic (16:0)	27.696	±1.153	27.518	±1.278	26.779	±1.671	26.962	± 1.404
d-7-hexadecenoic (16:1)	0.766	±0.114	0.808	±0.128	0.668	±0.092	0.669	±0.090
Palmitoleic (16:1)	2.090	±0.235	2.142	±0.258	0.694	±0.110	0.731	± 0.118
Stearic (18:0)	16.266	±1.803	16.712	±0.914	18.706	±1.554	18.842	± 2.183
Oleic (18:1)	6.518	±1.866	6.842	±1.700	5.262	±0.821	5.801	±1.273
Vaccenic (18:1)	4.472	±0.313	4.425	±0.359	3.261	±0.250	3.224	±0.180
Linoleic (18:2)	7.482	±0.707	7.851	±0.938	9.384	±1.365	8.912	±1.084
g-Linolenic (18:3)	0.082	±0.010	0.085	±0.008	0.056	±0.007	0.070	± 0.058
Linolenic (18:3)	0.548	±0.047	0.549	±0.061	0.411	±0.176	0.616	±1.036
Stearidonic (18:4)	n.d.		n.d.		n.d.		n.d.
Arachidic (20:0)	0.230	±0.128	0.203	±0.104	0.192	±0.109	0.210	±0.107
Eicosenoic (20:1)	0.176	±0.020	0.194	±0.029	0.145	±0.018	0.151	±0.021
Eicosadienoic (20:2)	0.283	±0.035	0.315	±0.058	0.803	±0.074	0.785	± 0.061
DHG-Linolenic (20:3)	0.925	±0.067	0.903	±0.078	0.761	±0.063	0.716	±0.048
Arachidonic (20:4)	11.976	±0.924	11.843	±0.794	10.756	±1.157	10.309	± 0.666
Eicosatrienoic (20:3)	n.d.		n.d.		n.d.		n.d.
Eicosatetraenoic (20:4)	n.d.		n.d.		n.d.		n.d.
Eicosapentaenoic (20:5)	0.076	±0.023	0.082	±0.008	0.055	±0.015	0.055	±0.014
Behenic (22:0)	0.346	±0.297	0.304	±0.219	0.181	±0.027	0.215	±0.109
Erucic (22:1)	0.094	±0.017	0.099	±0.027	0.056	±0.017	0.056	±0.016
Docosapentaenoic (22:5)	1.144	±0.132	1.214	±0.129	2.152	±0.163	2.095	±0.178
Docosahexaenoic (22:6)	16.665	±2.454	15.755	±1.726	18.221	±1.278	18.021	±1.479
Lignoceric (24:0)	0.499	±0.538	0.413	±0.383	0.262	±0.056	0.319	± 0.210
Nervonic (24:1)	0.501	±0.342	0.510	±0.301	0.258	±0.041	0.308	±0.134

*Values are depicted as mean ±standard deviation of total fatty acid fraction percentage; n = 18 per group. N.d., not detected. Muscle tissue from injured animals showed a significant influence (gray shade) on eicosenoic (ND, CvT) and DHG-linolenic acid (HFD, CvT) with p ≤ 0.05. Different depiction can be found in Supplementary Figure 1*.

### Fatty acid composition of triglyceride fraction

In order to determine changes in the amount of lipid metabolites, the triglyceride fraction was isolated from muscle tissue at all-time points, separated by thin-layer chromatography and analyzed by gas chromatography (Table 5). Stearidonic, eicosatrienoic and eicosatetraenoic acid could not be detected, whereas palmitic, oleic and linoleic acid reached at least 10% of total fatty acid fraction. Trauma showed a significant influence on docosahexaenoic acid (ND, CvT; *p* ≤ 0.05), while all other FAs remained unaffected (see Supplement 2.4).

**Table 5:** FA-composition of triglyceride fraction in muscle tissue.

	**Triglyceride fraction**							
	**Normal**				**Obese**			
	**Control**		**Trauma**		**Control**		**Trauma**	
Myristic (14:0)	2.089	±0.196	2.032	±0.158	1.189	±0.112	1.154	±0.076
Myristoleic (14:1)	0.162	±0.047	0.164	±0.046	0.052	±0.016	0.051	± 0.010
Palmitic (16:0)	26.202	±2.709	25.605	±1.803	20.582	±0.618	20.386	± 0.844
d-7-hexadecenoic (16:1)	0.819	±0.078	0.756	±0.115	0.768	±0.049	0.796	±0.049
Palmitoleic (16:1)	8.361	±1.645	8.379	±2.009	4.496	±0.807	4.518	± 0.494
Stearic (18:0)	8.178	±4.415	7.050	±1.731	6.026	±0.804	6.224	±1.271
Oleic (18:1)	37.411	±4.118	38.995	±2.252	40.837	±1.182	41.204	±1.700
Vaccenic (18:1)	3.313	±0.531	3.098	±0.345	3.043	±0.483	2.897	±0.270
Linoleic (18:2)	10.988	±2.090	11.357	±1.754	19.697	±0.723	19.555	± 0.788
g-Linolenic (18:3)	0.078	±0.021	0.071	±0.009	0.051	±0.006	0.053	± 0.009
Linolenic (18:3)	0.703	±0.269	0.639	±0.077	0.958	±0.126	0.908	±0.081
Stearidonic (18:4)	n.d.		n.d.		n.d.		n.d.
Arachidic (20:0)	0.174	±0.081	0.160	±0.042	0.106	±0.065	0.096	±0.024
Eicosenoic (20:1)	0.638	±0.086	0.667	±0.125	0.540	±0.090	0.529	± 0.046
Eicosadienoic (20:2)	0.193	±0.021	0.201	±0.039	0.544	±0.039	0.529	± 0.032
DHG-Linolenic (20:3)	0.126	±0.016	0.122	±0.020	0.168	±0.015	0.166	± 0.017
Arachidonic (20:4)	0.304	±0.058	0.333	±0.077	0.466	±0.066	0.459	± 0.054
Eicosatrienoic (20:3)	n.d.		n.d.		n.d.		n.d.
Eicosatetraenoic (20:4)	n.d.		n.d.		n.d.		n.d.
Eicosapentaenoic (20:5)	0.017	±0.004	0.018	±0.005	0.036	±0.007	0.033	±0.007
Behenic (22:0)	0.048	±0.012	0.049	±0.014	0.034	±0.007	0.035	±0.009
Erucic (22:1)	0.065	±0.019	0.063	±0.014	0.024	±0.006	0.024	±0.007
Docosapentaenoic (22:5)	0.068	±0.021	0.072	±0.021	0.101	±0.008	0.097	±0.010
Docosahexaenoic (22:6)	0.155	±0.019	0.190	±0.044	0.242	±0.029	0.239	±0.025
Lignoceric (24:0)	0.034	±0.012	0.034	±0.016	0.022	±0.008	0.022	± 0.008
Nervonic (24:1)	0.037	±0.014	0.032	±0.015	0.025	±0.015	0.028	±0.030

*Values are depicted as mean ± standard deviation of total fatty acid fraction percentage; n = 18 per group. N.d., not detected. Injury showed a significant influence (gray shade) on docosahexaenoic acid (ND, CvT) with p ≤ 0.05. Different depiction can be found in Supplementary Figure 2*.

### Expression levels of genes involved in FA metabolism

Genome wide profiling by microarray technology revealed that normal weight animals strongly responded to injury resulting in a total of 3829 differently expressed genes (DEG) in the whole time course after injury, whereas only 272 DEG were altered in obese animals, pointing to a weak response. Changes were detected in several pathways, including myeloid leukocyte migration, regulation of tumor necrosis factor production, CD4-positive, alpha-beta T cell differentiation, extracellular matrix organization, toll-like receptor (TLR) signaling pathway (own unpublished data), as well as FA metabolism, apoptosis, notch-, insulin-, and sonic hedgehog-signaling.

Analyzing fatty acid-related metabolism, 21 genes were identified to be significantly changed in at least one time point in muscle tissue of normal weight or obese animals (Table 6).

**Table 6:** Expression levels of genes involved in fatty acid metabolism determined by microarray analysis.

**Gene**	**Normal**					**Obese**				
	**1 h**	**6 h**	**24 h**	**72 h**	**192 h**	**1 h**	**6 h**	**24 h**	**72 h**	**192 h**
*Acot2*	1.03	−1.15	−1.09	−1.32	−1.35	1.55	1.19	**−2.07**	−1.03	1.03
***Alox5ap***	1.47	1.49	1.41	**8.78**	1.82	1.26	1.22	1.73	2.33	1.28
***Apobec1***	1.12	1.43	1.18	**8.46**	1.93	1.17	1.33	1.40	2.93	1.23
*Apobec2*	−1.12	1.01	1.12	**−1.83**	1.04	−1.08	−1.17	1.17	−1.36	−1.07
*Apobec3*	1.06	1.26	1.12	**3.66**	1.38	1.13	1.24	1.15	1.29	1.14
*Apoe*	1.20	1.01	1.13	**4.90**	**3.26**	−1.37	−1.13	1.11	2.20	1.24
*Elovl1*	1.27	1.17	1.11	**4.05**	1.46	−1.05	1.21	1.15	1.55	1.06
*Elovl5*	1.25	1.04	−1.13	**1.85**	1.36	−1.09	1.05	−1.07	1.10	1.15
*Fabp3*	−1.02	−1.10	**−1.91**	−1.47	−1.03	1.13	−1.07	−1.49	−1.25	1.14
*Fads1*	1.02	−1.09	1.02	**1.95**	1.06	−1.01	−1.21	−1.08	1.35	1.03
*Fads3*	1.41	1.04	1.34	**3.33**	**1.60**	−1.60	1.06	1.22	2.76	1.04
*Gltp*	1.01	−1.05	1.10	**2.74**	**1.60**	−1.13	−1.14	1.21	1.59	1.12
*Lcn2*	2.20	5.09	2.28	**2.54**	−1.12	5.65	1.91	5.93	1.68	1.07
*Ldlr*	1.18	1.69	−1.03	**1.76**	1.03	1.33	1.89	1.22	−1.04	1.00
*Lpl*	1.24	−1.18	**−1.91**	−1.42	−1.37	1.04	−1.12	−1.36	−1.24	1.13
*Lrp1*	1.25	1.24	−1.01	**2.79**	1.55	−1.07	1.01	1.22	1.50	1.14
*Lrp2bp*	−1.12	−1.11	1.21	**−3.27**	−1.46	−1.00	−1.25	−1.37	−1.58	−1.09
*Pltp*	1.43	−1.02	1.05	**5.32**	2.47	1.00	−1.03	−1.17	2.03	**1.33**
*Scd3*	1.05	1.01	1.09	**−1.21**	**−1.14**	1.06	−1.10	−1.05	−1.17	−1.00
*Srebf1*	−1.14	−1.03	−1.06	**−1.63**	1.19	1.03	−1.10	−1.00	1.04	1.14
*Srebf2*	1.04	1.08	1.09	**1.78**	1.30	1.08	1.14	**1.14**	1.05	1.07

*Significantly up-regulated genes (p ≤ 0.05) are shown in bold letters with background color (red = up-; green = down-regulated). Genes selected for validation are in bold. Description of genes and corresponding significance levels can be found in Supplement 3.1*.

DEGs were mostly seen 3 days post-injury for normal weight animals. Interestingly, genes that are modulated in response to injury after 3 d and/or 8 d in ND fed mice do not show significant alterations under obese conditions. Generally, expression levels were relatively low due to the focus on the influence of trauma vs. control. For significance levels see Supplement 3.1.

DEGs derived from muscle tissue of injured and control normal weight and obese mice were used for pathway analysis. Four major players were identified, namely cholesterol homeostasis (41 hits; 67.21%), fatty acid biosynthetic process (11 hits; 18.03%), fatty acid elongation (7 hits; 11.48%) and acyl-CoA metabolic process (2 hits; 3.28%) with high confidence and at least three matches to the data base. Pathway analysis was carried out with ClueGO v2.3.3, a plug-in for Cytoscape.

**Figure 4:**
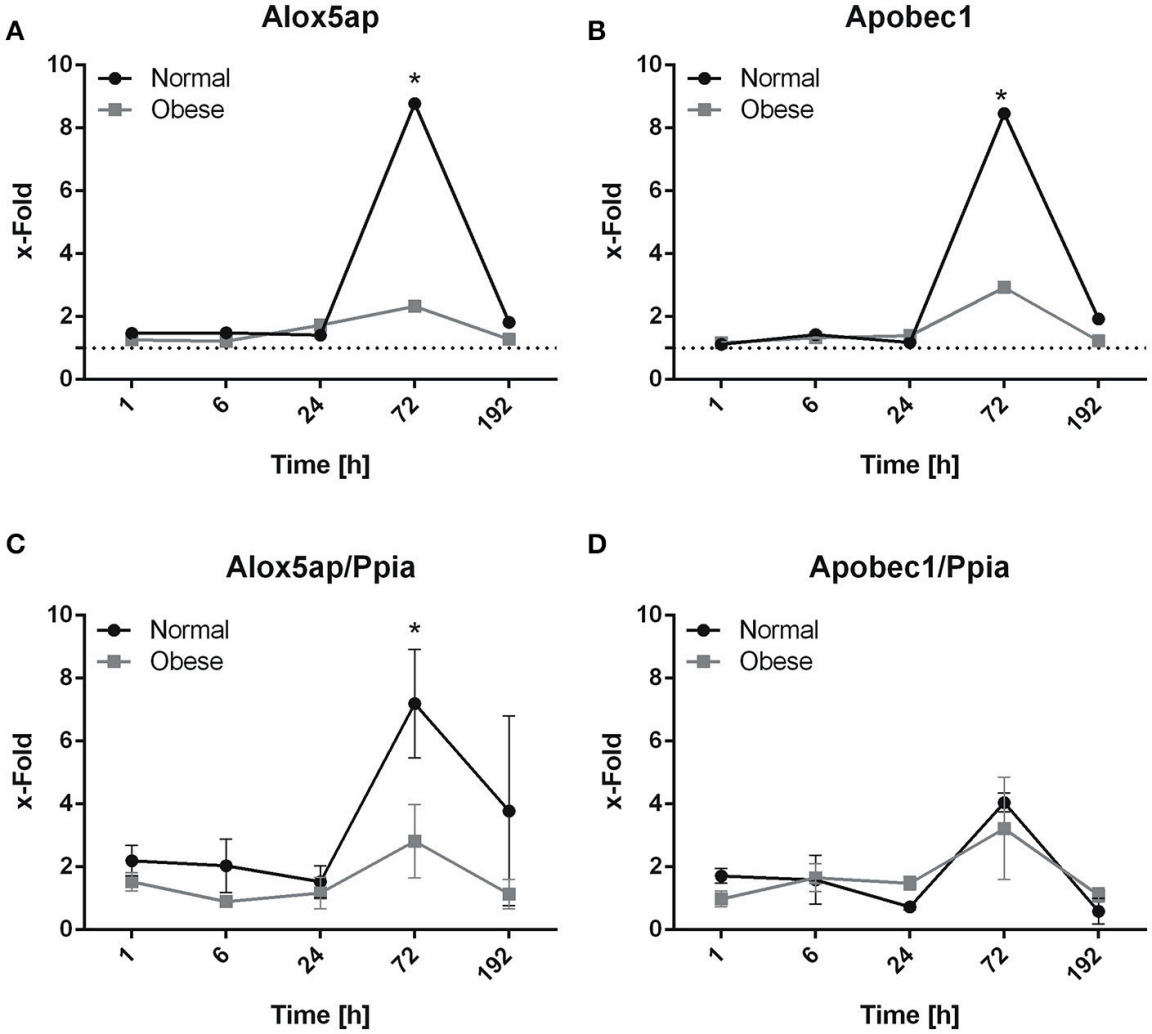
Expression levels of *Alox5ap* **(A)** and *Apobec1* **(B)** as determined by microarray analysis. Ticked line(s) at 1 represents basal level. ^*^p ≤ 0.05. Expression levels of *Alox5ap* **(C)** and *Apobec1* **(D)** quantified by qPCR. X-fold values ±SEM were determined by normalization on corresponding controls of each time point and diet. Genes show the highest differential expression 3 days post-injury, while obese animals show a lack of response to trauma, overall indicating a direct influence of injury. *Apobec1* **(D)** shows a unidirectional response despite the higher SEM. Both genes show a similar behavior in microarray and qPCR. Significance of qPCR was determined using ANOVA.

Screening of data revealed strong up-regulation of *Alox5ap* (GeneCards(R), 2017a) and *Apobec1* (GeneCards(R), 2017c) three days post-injury. Gene expression levels derived from microarray analysis of *Alox5ap* (Figure 4A), and *Apobec1* (Figure 4B) show a significant increase resulting in a peak 72 h post-injury. A different response is imminent between female normal weight and obese C57BL/6J mice, where obese animals show a hampered response. Whereas validation by qPCR analysis revealed a significant up-regulation of *Alox5ap* in normal weight mice compared to obese mice 72 h post-injury (Figure 4C), the differences in *Apobec1* expression levels could not be confirmed (Figure 4D).

In order to assess the influence of obesity on regeneration-relevant pathways, the expression levels of genes associated with apoptosis, notch-, insulin-, and sonic hedgehog-signaling were evaluated. This analysis revealed significantly altered expression levels of 4 genes involved in notch, 32 genes in insulin, 8 genes in sonic hedgehog, and 35 genes in apoptosis in at least one time point in muscle tissue of normal weight or obese animals (Table 7). DEG were mostly seen 3 days post-injury for normal weight, whereas obese animals showed a general lack of response (for significance levels, see Supplement 3.2).

**Table 7:** Expression levels of genes associated with notch, insulin, sonic hedgehog (Shh) and apoptosis.

**Gene**	**Trauma vs. Control**										
	**Normal**					**Obese**					
	**1 h**	**6 h**	**24 h**	**3 d**	**8 d**	**1 h**	**6 h**	**24 h**	**3 d**	**8 d**	
**Notch**											
*Adam17*	1.19	1.11	**−1.22**	**1.94**	1.06	1.11	1.13	−1.20	1.12	1.16	
*Adam19*	1.22	1.09	−1.09	**2.12**	**1.80**	1.18	1.03	1.04	1.23	1.09	
*Lfng*	−1.03	1.09	1.20	**2.38**	1.49	−1.26	−1.09	**1.35**	1.65	1.15	
*Notch2*	1.33	1.49	1.00	**2.63**	1.50	1.13	1.13	1.24	1.45	1.19	
**Insulin**											
*Akt1*	−1.02	1.16	1.20	**2.37**	1.49	1.01	1.09	1.20	1.07	1.00	
*Foxo4*	−1.02	−1.08	−1.09	**−2.02**	**−1.65**	−1.08	−1.09	−1.05	−1.01	−1.02	
*Fyn*	1.38	1.05	−1.17	**2.63**	**2.21**	−1.05	1.10	−1.06	1.08	1.30	
*Inpp5d*	1.37	**1.33**	1.13	**5.22**	1.50	1.08	1.20	1.21	1.95	1.07	
*Pdk1*	−1.01	1.05	**−1.12**	**−1.56**	**−1.30**	1.11	−1.10	**−1.20**	−1.15	1.09	
*Pdk2*	**−1.13**	−1.09	1.06	**−2.08**	**−1.31**	−1.01	−1.09	**−1.06**	−1.13	−1.11	
*Pdk3*	1.07	1.11	1.15	**2.86**	1.33	−1.12	1.06	1.01	1.02	−1.01	
*Pdk4*	1.10	1.08	−1.21	−1.07	−1.25	1.16	1.14	**−1.91**	−1.08	1.04	
*Ptpn1*	1.19	1.62	1.23	**1.99**	1.07	1.03	1.24	1.15	1.20	−1.03	
*Raf1*	−1.19	−1.16	−1.10	**−1.50**	−1.35	−1.19	−1.16	−1.03	−1.07	−1.07	
*Sgk3*	1.14	1.10	**−1.24**	**1.83**	−1.00	**1.21**	1.06	−1.17	−1.08	1.10	
**Sonic hedgehog**											
*Glis2*	1.20	1.08	**−1.16**	**1.56**	**1.33**	1.07	−1.06	1.02	1.07	−1.00	
*Myc*	**2.58**	2.67	2.06	2.66	1.30	**3.62**	2.27	1.38	1.04	−1.36	
**Apoptosis**											
*Bak1*	−1.32	1.01	**1.26**	**1.99**	**1.44**	−1.15	−1.19	1.14	1.15	1.04	
*Bax*	−1.07	1.18	1.07	**1.90**	1.20	−1.02	1.01	−1.04	1.05	1.01	
*Bid*	1.08	−1.24	1.08	**1.61**	1.26	1.04	1.01	1.06	1.15	1.04	
*Capn2*	1.17	**1.21**	−1.06	**2.16**	1.30	−1.07	1.12	1.02	1.26	1.20	
*Capn6*	**−1.18**	−1.34	**1.32**	**7.27**	3.56	−1.00	−1.24	−1.09	1.52	1.09	
*Capn7*	−1.01	−1.02	−1.12	**−1.62**	**−1.40**	−1.05	1.02	−1.09	−1.06	−1.00	
*Casp3*	−1.00	−1.05	1.08	**3.13**	**1.33**	−1.02	1.14	1.10	1.27	1.02	
*Casp6*	1.02	−1.26	−1.10	**2.26**	1.43	−1.16	1.22	−1.01	1.16	1.12	
*Casp8*	1.31	1.40	1.46	**5.56**	1.58	1.09	1.49	1.38	1.92	1.12	
*Cdk1*	−1.09	−1.07	1.05	**2.96**	1.17	1.00	−1.06	1.02	1.25	1.02	
*Ikbip*	1.02	1.01	1.08	**1.60**	1.17	−1.01	−1.01	1.14	1.13	−1.03	
*Junb*	1.86	2.07	−1.02	**1.54**	1.27	3.00	1.58	**1.14**	1.10	1.02	
*Jund*	**1.23**	1.19	1.06	1.17	1.28	**1.63**	−1.13	−1.05	−1.01	−1.06	
*Nfkb1*	1.25	1.25	−1.00	**1.67**	1.05	1.15	1.17	1.03	1.06	−1.01	
*Nfkbid*	1.40	1.05	1.00	1.06	1.05	**1.73**	1.10	1.06	1.05	1.04	
*Nfkbie*	1.23	1.02	1.09	**2.38**	1.29	**1.24**	1.04	1.06	1.18	1.19	
*Nfkbiz*	2.08	1.41	1.02	**1.20**	1.02	**2.97**	1.33	−1.05	1.10	1.07	
*Ripk1*	**1.43**	**1.36**	−1.01	**2.20**	**1.31**	1.73	1.27	1.09	1.28	1.10	
*Rock1*	1.38	1.28	−1.25	**2.16**	1.09	1.03	1.29	−1.00	1.03	1.28	
*Trp53*	−1.07	1.04	**1.59**	2.46	**1.33**	1.07	1.28	1.06	1.16	−1.17	
*Trp53inp1*	1.18	1.27	−1.42	1.00	1.06	1.01	**−1.66**	−1.27	−1.08	1.05	
**Shared genes**											Shared with
*Akt1s1*	−1.19	1.01	1.04	**−1.61**	**−1.25**	−1.06	−1.12	−1.09	−1.01	−1.01	Apoptosis Insulin
*Akt3*	**1.31**	1.04	**−1.79**	**1.86**	1.39	1.00	1.10	−1.38	−1.09	1.32	
*Mapk3*	−1.10	−1.10	1.10	**1.88**	1.18	**−1.26**	−1.12	−1.01	1.14	−1.03	
*Pik3c2a*	1.21	1.10	−1.03	**1.61**	1.12	1.09	1.19	1.05	1.05	1.20	
*Pik3cg*	**1.29**	1.12	−1.15	**2.54**	1.36	1.10	1.17	1.07	1.63	1.08	
*Pik3ip1*	1.09	−1.31	**−1.44**	**−2.06**	**−1.57**	−1.13	−1.26	−1.37	−1.01	1.01	
*Pik3r5*	1.65	1.27	1.07	**3.01**	1.44	1.40	1.45	1.20	1.68	1.11	
*Prkca*	−1.05	−1.11	−1.03	**−1.70**	**−1.35**	1.02	−1.10	**−1.18**	1.04	1.04	
*Prkcb*	1.08	1.02	1.05	**2.18**	**1.26**	1.04	−1.12	1.07	1.22	1.07	
*Prkcd*	1.19	**1.43**	−1.13	**2.96**	**1.28**	1.03	1.36	1.06	1.73	1.10	
*Prkcdbp*	1.13	1.14	1.27	**2.40**	1.41	−1.20	1.03	1.30	1.48	1.00	
*Prkch*	1.02	1.16	−1.11	**1.71**	1.06	1.21	1.10	1.04	1.06	1.14	
*Prkci*	1.27	1.12	−1.04	**1.82**	1.17	1.04	1.23	1.08	1.11	1.10	
*Prkcq*	1.05	−1.11	−1.10	**−1.51**	1.03	1.10	−1.08	−1.12	−1.05	−1.06	
*Prkcsh*	1.01	−1.05	−1.03	**1.68**	−1.02	−1.06	−1.01	−1.13	1.01	−1.05	
*Prkaa1*	1.12	1.07	−1.17	**1.51**	1.30	1.03	1.13	−1.13	−1.00	1.07	Insulin Shh
*Prkaa2*	−1.02	−1.09	1.06	**−2.32**	**−1.54**	−1.10	−1.13	1.00	1.05	−1.11	
*Prkab2*	−1.08	−1.15	−1.01	**−2.33**	**−1.80**	−1.18	−1.08	1.03	1.20	−1.17	
*Prkag1*	−1.09	−1.04	−1.04	**−1.52**	−1.29	−1.00	−1.05	1.00	**−1.25**	−1.07	
*Prkag3*	−1.34	−1.37	1.53	**−2.86**	−1.20	−1.41	−1.30	1.56	1.29	−1.02	
*Prkar2a*	−1.25	−1.14	1.02	**−1.73**	**−1.38**	−1.21	−1.11	1.08	1.04	−1.04	

*The shared section includes genes involved in at least 2 pathways. Significantly up-regulated genes (p ≤ 0.05) are shown in bold letters with background color (red = up-; green = down-regulated). Description of genes and corresponding significance levels can be found in Supplement 3.2*.

## Discussion

Increasing evidence suggests that obesity strongly impairs muscle regeneration due to chronic inflammation and excessive accumulation of lipids in both, adipose and non-adipose tissues (Akhmedov and Berdeaux, 2013). Here, we compared the influence of obesity related factors before and after trauma focusing on fatty acid and gene expression profiles after induction of a blunt trauma to *extensor iliotibialis anticus* on the left hind limb of normal weight and obese female C57BL/6J mice.

Feeding female C57BL/6J mice with a defined 60% kcal high fat diet resulted in the obesity phenotype where the body weight was significantly increased when compared to their normal calorie diet counterparts. The greater standard deviation in the weight of obese mice is possibly the result of individual differences in metabolism and physical activity of normal weight and obese mice, which also has been reported for humans (Montague et al., 1997; Ali and Crowther, 2009; Bryan et al., 2011; Brady, 2016). In contrast to the diet, induction of a blunt muscle trauma did not result in any significant changes in body weight of normal weight and obese mice post-injury. Therefore, we can exclude that possible differences in movement of normal weight and obese mice prominently influence their body weight post-injury. No significant differences in force input into the muscles of normal and obese mice were observed (own unpublished data), as well as no distinct differences in hematoma, and edema formation could be detected, whereas morphology showed an unfinished regeneration for obese mice 8 d post-injury. Analysis of FA content in muscles of normal weight and obese mice surprisingly revealed, that muscle tissue from normal weight animals showed higher SFA content in the TG fraction, indicating that higher amounts of the saturated FAs are stored. This is especially noteworthy as obese animals have a higher proportion of SFAs in their diets (22.71% ND vs. 32.02% HFD), thus making an inverted result more expected. PL fraction of muscle tissue from normal weight mice showed a higher MUFA content, while PUFAs were higher in obese mice. As stated in the content chart by the manufacturer, ND diet contains a lower ratio of MUFA when compared to HFD (29.82 vs. 35.95%), whereas PUFAs were higher in ND (47.48 vs. 32.02%). While normal weight animals have a lower uptake of MUFAs within their diet, they surprisingly show a higher MUFA content in skeletal muscle. In contrast, PUFA ratio was higher in the normal diet, while exhibiting a lowered content in the muscle of the ND group. This is presumably dependent on the variations in total amount in grams for SFA, MUFA, and PUFA for the different diets, wherein the HFD showed overall higher amounts (Ulman, 2011). Furthermore, these differences could be explained by the limitation in total FA uptake by the 10% kcal fat diet, which leads to increased synthesis of saturated FA for repositories. Obese animals had most of the FAs and its precursors abundantly present in the 60% kcal fat diet, making the excessive storage and synthesis, at least in parts, unnecessary. This means, that although obese animals were able to consume higher total amounts of FA, they were not required to store as much of these FAs as they had plenty present within their diet. On the other hand, normal weight animals had to build up repositories to accommodate for potential starving periods. Additionally, lipid overload has been recognized as one important factor leading to muscle mass reduction, which contributes to impaired muscle regeneration after injury (Tamilarasan et al., 2012; Akhmedov and Berdeaux, 2013). Thus, the FA ratios in normal weight mice correlate to a healthy individual and seem to lead to a regular response to injury. In contrast, obese animals are known to show a delayed reply to injury (Nguyen et al., 2011; Woo et al., 2011; Akhmedov and Berdeaux, 2013; D'Souza et al., 2015), which also derives from variation in FA content in skeletal muscle. Although we observed several differences in FA ratios, no significance was detectable upon combining all increments for Fisher's exact test and odds ratio. Consequently, cumulated FA content of skeletal muscle is independent of injury, while the diet does exhibit slightly higher odds of increased FA content. The presented data implicates a lack of response in obese mice due to differences in fatty acid composition of skeletal muscle. In this context, a closer look at significantly altered FAs upon injury is necessary.

Blunt muscle injury resulted in changes of the levels of two fatty acids in the phospholipid [eicosenoic (ND, CvT) and DHG-linolenic acid (HFD, CvT); *p* ≤ 0.05] and of one FA in the triglyceride (docosahexaenoic acid (ND, CvT); *p* ≤ 0.05) fraction.

Muscle injury seems to trigger the elongation from oleic (OA; 18:1) to eicosenoic acid (EA; 20:1) in PL fraction in control compared to injured normal weight mice. This assumption is supported by our gene expression data wherein an increase in *Elovl1/3* is detectable, making injury a potential triggering factor of EA synthesis. It is well established that omega fatty acids are important mediators for growth and development (Abood et al., 2014). Despite this, there is little evidence of the exact mechanisms of EA in the body. Nevertheless, our data show decreased levels of EA in obese mice in PL and TG fraction, suggesting a correlation with impaired muscle regeneration after injury in obese animals. Interestingly, the precursor OA shows lower levels in PL fraction but higher levels in TG fraction for obese mice, indicating an impaired incorporation into cell membranes, while storing higher amounts. OA is known to play a role in countering TNF-α mediated functions regarding insulin (Vassiliou et al., 2009). This may enhance the negative outcome for obese individuals due to improper mediation of signals.

The reduction of DHG-linolenic acid (DHGL; 20:3) in control compared to injured obese mice in PL fraction might partially result from the excessive availability of precursors in the diet (Gajda, 2017) leading to a redundant depository. Additionally, omega-3-fatty acids are integral parts of the cell membrane (Valentine and Valentine, 2004; Surette, 2008). DHGL is a precursor for the synthesis of arachidonic acid (AA), a FA involved in inflammatory responses. AA is a crucial cell membrane component of cells involved in modulating the magnitude and period of inflammatory responses. It exhibits its functions via the synthesis of mostly pro-inflammatory eicosanoids (Calder, 2010; Markworth and Cameron-Smith, 2012; Yashiro et al., 2016). Our phospholipid fraction data show, that the amount of DHGL is negatively influenced by injury in obese mice, while normal weight animals do not show a significant impact, despite higher content. Thus, the response of obese mice might be an increased synthesis of AA from DHGL. This is reflected in the triglyceride fraction of skeletal muscle, where a higher amount of stored DHGL and AA is observed.

The increase in docosahexaenoic acid (DHA; 22:6) in control versus injured normal weight mice in TG fraction may be a direct response to injury. DHA is known to have an anti-inflammatory effect by changing cell membrane composition of cells involved in inflammatory response, while directly affecting pro-inflammatory cytokines as well (Calder, 2010; Mullen et al., 2010; Prostek et al., 2014). An appropriate effect seems to be mediated by a slightly increased DHA content, whereas high levels of DHA are able to affect viability by means of apoptosis as described previously (Sun et al., 2017). Although being tested in a cell culture model, the results show the potential induction of the p53, MAPK, TNF, PI3K/Akt and NF-κB pathways by DHA (Sun et al., 2017). The increased levels of DHA and its precursor docosapentaenoic acid may propagate pro-apoptotic effects in obese mice that showed higher levels of these FAs when compared to normal weight mice, potentially contributing to an impaired regeneration after injury.

Animals were grouped per time point, which further required the direct comparison of trauma vs. control in order to generate a time lapse for our gene expression analysis. Insignificant values were included to assess changes as a whole without excluding masked hits. Normal weight mice showed a strong response to injury in FA-related genes, thus further confirming the lack of response in obese mice compared to injury (Akhmedov and Berdeaux, 2013; D'Souza et al., 2015). Interestingly, while screening FA-related genes, we noted that genes involved in lipoprotein metabolism (*apoE, LDLR, Lrp1, Pltp, Srebf2, and Apobec*), in the synthesis of membrane lipids (*Elovl1, Gltp, Elovl5, Fads1, Fads3*), and lipid mediators (*Alox5ap*) were differentially expressed. A striking candidate among these is *Alox5ap*, encoding the activating protein for the gene product of *Alox5*, which is involved in leukotriene synthesis. Leukotrienes are derivatives of arachidonic acid (AA) and responsible for inflammatory responses, e.g., in human muscle (Markworth and Cameron-Smith, 2012; GeneCards(R), 2017a). Our data showed that AA content in mouse skeletal muscle was lower in PL and higher in TG fraction in muscle tissue of obese animals. Omega-3-PUFAs are commonly incorporated into cell membranes to modify membrane viscosity, thus influencing cell-cell interactions (Ander et al., 2003; Balogun et al., 2013; Duivenvoorde et al., 2015; Yang et al., 2017). Omega-6-PUFAs are also known to be potent lipid mediators in the modification of an inflammatory response (Patterson et al., 2012). The higher content of AA in PL fraction could indicate the regular function in normal weight animals, while the decreased levels of AA in obese mice lead to a limited anti-inflammatory response of, e.g., immune cells (Calder, 2010). TG fraction shows inverse results for both, normal weight and obese mice. However, obese animals are not able to take advantage of a higher AA deposit, potentially by excessive lipid overload (Tamilarasan et al., 2012). This assumption is reflected in the hampered *Alox5ap* expression levels of obese animals. *Apobec1* is also involved in lipoprotein metabolism and considered a sign for immune cell infiltration due to its association with apolipoprotein B, which is incorporated in low density lipoproteins (GeneCards(R), 2017b,c). Up-regulation of *Apobec1* correlates to a proper response in normal weight mice, while obese animals do show a similar tendency.

Evaluation of pathways involved in cell proliferation, differentiation and removal of tissue debris were analyzed as well. Notch regulates cell-fate determination during development and tissue homeostasis (Bentzinger et al., 2012) and was mostly upregulated in injured muscles of normal weight animals, whereas obese mice showed a lack of expression within this pathway, thus hampering with regeneration. The insulin pathway regulates energy consumption by glucose- and lipid-metabolism (Guilherme et al., 2008) and shows a variation of up- and down-regulation for both groups, whereas obese animals generally show a down-regulation in expression levels. Three days post-injury is a crucial time point for normal weight mice as they show a stronger activity within this pathway, as a lot of energy is required for the regeneration process. In contrast, obese animals apparently do not require a high activity, probably due to their increased uptake of FAs, resulting in an impaired pathway. Sonic hedgehog is responsible for embryonic development and tissue maintenance (Bentzinger et al., 2012). In general, only little activity is seen, whereas the observed upregulation may account for the tissue maintenance of the injured muscle tissue in normal weight animals. Induction of apoptosis results in controlled cell death, wherein caspases play crucial roles (Darby et al., 2016). This pathway is mostly silent in obese mice, while their normal weight counterparts show a distinct activity mostly 3 days post-injury, meaning that the removal of damaged tissue is functioning properly. Obese animals lack this activity at the evaluated time points, resulting in either an impaired or delayed response.

In summary, we were able to successfully apply an improved drop tower-device for blunt muscle injury in normal weight and obese mice. The high fat diet changed the composition of FA in the skeletal hind-limb muscle of female C57BL/6J mice. Gas chromatographic analysis revealed changes in SFA, MUFA, and PUFA content for phospholipid and triglyceride fractions. Furthermore, diet and injury were able to significantly alter eicosenoic (ND; CvT) and DHG-linolenic acid (HFD, CvT) in phospholipid and docosahexaenoic acid (ND, CvT) in triglyceride fraction. We also identified 3 days as the most important time point in response to injury for fatty acid related genes in normal weight mice, while obese animals are likely to be delayed. Pathway analysis of differentially expressed genes showed cholesterol homeostasis, fatty acid biosynthetic process, fatty acid elongation and acyl-CoA metabolic process with high confidence. This outcome led to the identification of *Alox5ap* and *Apobec1* for further analysis, where we were able to show similar expression profiles, albeit significant differences between normal weight and obese were observed only at 3 d post-injury for *Alox5ap*. Nevertheless, both genes indicate a regular function of the synthesis of lipid mediators, lipid metabolism and immune function for normal weight mice. Considering this, the change in fatty acid metabolism may directly contribute to the impaired muscle regeneration in obese animals due to altered FA ratios, FAs and gene expression levels. In addition, notch-, insulin-, sonic hedgehog-, and apoptosis-signaling are impaired in obese mice, while normal weight animals showed major influences 3 d post-injury.

## Author contributions

UK and MW designed the experiments. UK and MW received funding and supervised the study. PX, LL, and J-UW performed animal experiments and collected tissue. KT and J-UW prepared samples for GC analysis. LS, KT, and J-UW evaluated GC data. AS, MJ, PG, and J-UW designed the approach and R script for microarray evaluation. LS and J-UW evaluated microarray data. The qPCR was performed by J-UW. UK, MW, PX and J-UW wrote the paper with input of the other authors. All authors read the final manuscript and approved it.

### Conflict of interest statement

The authors declare that the research was conducted in the absence of any commercial or financial relationships that could be construed as a potential conflict of interest.

## Supplementary material

Supplementary Figure 1 Fatty acid content in phospholipid fraction. Values are depicted as mean ± standard deviation.

Supplementary Figure 2 Fatty acid content in triglyceride fraction. Values are depicted as mean ± standard deviation.

Supplement 1.1 Contingency tables of FA ratios used for Fisher's exact test and odds ratio including calculated results.

Supplement 1.2 List of packages used with RStudio 0.99.903 and R 3.2.5.

Supplement 2.1 Significance levels of FA-composition in phospholipid fraction in muscle tissue; *n* = 18 per group. Statistical analysis by two-sided homoscedastic *t*-test. ^*^Indicates *p* ≤ 0.01, ^**^Indicates *p* ≤ 0.05.

Supplement 2.2 Fatty acid content in phospholipid fraction from 1 h to 21 d post-injury for female normal weight C57BL/6J mice. N.d., not detected.

Supplement 2.3 Fatty acid content in phospholipid fraction from 1 h to 21 d post-injury for female obese C57BL/6J mice. N.d., not detected.

Supplement 2.4 Significance levels of FA-composition in triglyceride fraction in muscle tissue; *n* = 18 per group. Statistical analysis by two-sided homoscedastic *t*-test. ^*^Indicates *p* ≤ 0.01, ^**^Indicates *p* ≤ 0.05.

Supplement 2.5 Fatty acid content in triglyceride fraction from 1h to 21d post-injury for female normal weight C57BL/6J mice. N.d., not detected.

Supplement 2.6 Fatty acid content in triglyceride fraction from 1h to 21d post-injury for female obese C57BL/6J mice. N.d., not detected.

Supplement 3.1 ID's, gene name, description and significance levels of fat metabolism microarray analysis. Red = *p* ≤ 0.05; Green = *p* ≤ 0.01.

Supplement 3.2 ID's, gene name, description and significance levels of Notch, Insulin, Sonic hedgehog and Apoptosis microarray analysis. Red = *p* ≤ 0.05; Green = *p* ≤ 0.01.
