# Supplementary material for: Comparison of Fatty Acid and Gene Profiles in Skeletal Muscle in Normal and Obese C57BL/6J Mice before and after Blunt Muscle Injury
Source: Front Physiol. 2018 Jan 30;9:19. doi: 10.3389/fphys.2018.00019 (PMC5797686; doi:10.3389/fphys.2018.00019)
Supplement: Supplement 1.1 — Contingency tables of FA ratios used for Fisher's exact test and odds ratio including calculated results. [file Supplement1.1.DOCX]

Supplementary Material

Comparison of fatty acid and gene profiles in skeletal muscle in normal and obese C57BL/6J mice before and after blunt muscle injury

Jens-Uwe Werner^1†^, Klaus Tödter^2†^, Pengfei Xu^1^, Lydia Lockhart^1^, Markus Jähnert^3^, Pascal Gottmann^3^, Annette Schürmann^3^, Ludger Scheja^2^, Martin Wabitsch^4,^*, Uwe Knippschild^1,^*

* Correspondence: Prof. Dr. Martin Wabitsch, Ulm University Hospital for Pediatrics and Adolescent Medicine, Division of Pediatric Endocrinology and Diabetes, Eythstraße 24, 89075 Ulm, Germany, martin.wabitsch@uniklinik-ulm.de and Prof. Dr. Uwe Knippschild, Ulm University Hospital, Department of General and Visceral Surgery, Albert-Einstein-Allee 23, 89081 Ulm, Germany, uwe.knippschild@uniklinik-ulm.de

Supplement 1.1: Contingency tables of FA ratios used for Fisher’s exact test and odds ratio including calculated results.

|  | **Phospholipid fraction** | | | | | | |
| --- | --- | --- | --- | --- | --- | --- | --- |
| **PUFA** | **Control** | **Trauma** |  | **Odds Ratio** | **Quantile (2.5%)** | **Quantile (97.5%)** | **Fisher's Exact test** |
| **Normal** | 39.180 | 38.597 |  | 0.977 | 0.505 | 1.89 | 1 |
| **Obese** | 42.598 | 41.581 |  |  |  |  |  |
|  |  |  |  |  |  |  |  |
| **MUFA** | **Control** | **Trauma** |  | **Odds Ratio** | **Quantile (2.5%)** | **Quantile (97.5%)** | **Fisher's Exact test** |
| **Normal** | 14.616 | 15.019 |  | 1.098 | 0.313 | 3.881 | 1 |
| **Obese** | 10.345 | 10.940 |  |  |  |  |  |
|  |  |  |  |  |  |  |  |
| **SFA** | **Control** | **Trauma** |  | **Odds Ratio** | **Quantile (2.5%)** | **Quantile (97.5%)** | **Fisher's Exact test** |
| **Normal** | 46.142 | 46.309 |  | 1 | 0.541 | 1.85 | 1 |
| **Obese** | 46.898 | 47.320 |  |  |  |  |  |
|  | **Triglyceride fraction** | | | | | | |
| **PUFA** | **Control** | **Trauma** |  | **Odds Ratio** | **Quantile (2.5%)** | **Quantile (97.5%)** | **Fisher's Exact test** |
| **Normal** | 12.630 | 13.002 |  | 1 | 0.34 | 2.945 | 1 |
| **Obese** | 22.264 | 22.039 |  |  |  |  |  |
|  |  |  |  |  |  |  |  |
| **MUFA** | **Control** | **Trauma** |  | **Odds Ratio** | **Quantile (2.5%)** | **Quantile (97.5%)** | **Fisher's Exact test** |
| **Normal** | 50.805 | 52.155 |  | 0.981 | 0.545 | 1.764 | 1 |
| **Obese** | 49.785 | 50.047 |  |  |  |  |  |
|  |  |  |  |  |  |  |  |
| **SFA** | **Control** | **Trauma** |  | **Odds Ratio** | **Quantile (2.5%)** | **Quantile (97.5%)** | **Fisher's Exact test** |
| **Normal** | 36.725 | 34.930 |  | 1.057 | 0.496 | 2.254 | 1 |
| **Obese** | 27.959 | 27.916 |  |  |  |  |  |
