# Supplementary material for: Comparison of Fatty Acid and Gene Profiles in Skeletal Muscle in Normal and Obese C57BL/6J Mice before and after Blunt Muscle Injury
Source: Front Physiol. 2018 Jan 30;9:19. doi: 10.3389/fphys.2018.00019 (PMC5797686; doi:10.3389/fphys.2018.00019)
Supplement: Supplement 1.2 — List of packages used with RStudio 0.99.903 and R 3.2.5. [file Supplement1.2.DOCX]

Supplementary Material

Comparison of fatty acid and gene profiles in skeletal muscle in normal and obese C57BL/6J mice before and after blunt muscle injury

Jens-Uwe Werner^1†^, Klaus Tödter^2†^, Pengfei Xu^1^, Lydia Lockhart^1^, Markus Jähnert^3^, Pascal Gottmann^3^, Annette Schürmann^3^, Ludger Scheja^2^, Martin Wabitsch^4,^*, Uwe Knippschild^1,^*

* Correspondence: Prof. Dr. Martin Wabitsch, Ulm University Hospital for Pediatrics and Adolescent Medicine, Division of Pediatric Endocrinology and Diabetes, Eythstraße 24, 89075 Ulm, Germany, martin.wabitsch@uniklinik-ulm.de and Prof. Dr. Uwe Knippschild, Ulm University Hospital, Department of General and Visceral Surgery, Albert-Einstein-Allee 23, 89081 Ulm, Germany, uwe.knippschild@uniklinik-ulm.de

Supplement 1.2: List of packages used with RStudio 0.99.903 and R 3.2.5.

| **Package** | **Version** | **Citation** |
| --- | --- | --- |
| questionr | 0.6.1 | Julien Barnier, François Briatte and Joseph Larmarange (2017). questionr: Functions to Make Surveys Processing Easier. R package version 0.6.1. <http://CRAN.R-project.org/package=questionr> |
| affy | 1.52.0 | Gautier, L., Cope, L., Bolstad, B. M., and Irizarry, R. A. 2004. affy---analysis of Affymetrix GeneChip data at the probe level. Bioinformatics 20, 3 (Feb. 2004), 307-315. |
| mogene10sttranscriptcluster.db | 8.5.0 | James W. MacDonald (2016). mogene10sttranscriptcluster.db: Affymetrix mogene10 annotation data (chip mogene10sttranscriptcluster). R package version 8.5.0. |
| org.Mm.eg.db | 3.4.0 | Marc Carlson (2016). org.Mm.eg.db: Genome wide annotation for Mouse. R package version 3.4.0. |
| AnnotationDbi | 1.36.0 | Herve Pages, Marc Carlson, Seth Falcon and Nianhua Li (2016). AnnotationDbi: Annotation Database Interface. R package version 1.36.0. |
| Iranges | 2.8.1 | Lawrence M, Huber W, Pagès H, Aboyoun P, Carlson M, et al. (2013) Software for Computing and Annotating Genomic Ranges. PLoS Comput Biol 9(8): e1003118. doi:10.1371/journal.pcbi.1003118 |
| S4Vectors | 0.12.0 | H. Pagès, M. Lawrence and P. Aboyoun (2016). S4Vectors: S4 implementation of vectors and lists. R package version 0.12.0. |
| Biobase | 2.34.0 | Orchestrating high-throughput genomic analysis with Bioconductor. W. Huber, V.J. Carey, R. Gentleman, ..., M. Morgan Nature Methods, 2015:12, 115. |
| BiocGenerics | 0.20.0 | Orchestrating high-throughput genomic analysis with Bioconductor. W. Huber, V.J. Carey, R. Gentleman, ..., M. Morgan Nature Methods, 2015:12, 115. |
| preprocessCore | 1.36.0 | Benjamin Milo Bolstad (2016). preprocessCore: A collection of pre-processing functions. R package version 1.36.0. https://github.com/bmbolstad/preprocessCore |
| calibrate | 1.7.2 | Jan Graffelman (2013). calibrate: Calibration of Scatterplot and Biplot Axes. R package version 1.7.2. https://CRAN.R-project.org/package=calibrate |
| MASS | 7.3-45 | Venables, W. N. & Ripley, B. D. (2002) Modern Applied Statistics with S. Fourth Edition. Springer, New York. ISBN 0-387-95457-0 |
| gtools | 3.5.0 | Gregory R. Warnes, Ben Bolker and Thomas Lumley (2015). gtools: Various R Programming Tools. R package version 3.5.0. https://CRAN.R-project.org/package=gtools |
| missMDA | 1.10 | Julie Josse, Francois Husson (2016). missMDA: A Package for Handling Missing Values in Multivariate Data Analysis. Journal of Statistical Software, 70(1), 1-31. doi:10.18637/jss.v070.i01 |
| pvclust | 2.0-0 | Ryota Suzuki and Hidetoshi Shimodaira (2015). pvclust: Hierarchical Clustering with P-Values via Multiscale Bootstrap Resampling. R package version 2.0-0. https://CRAN.R-project.org/package=pvclust |
| FactoMineR | 1.33 | Sebastien Le, Julie Josse, Francois Husson (2008). FactoMineR: An R Package for Multivariate Analysis. Journal of Statistical Software, 25(1), 1-18. 10.18637/jss.v025.i01 |
| gplots | 3.0.1 | Gregory R. Warnes, Ben Bolker, Lodewijk Bonebakker, Robert Gentleman, Wolfgang Huber Andy Liaw, Thomas Lumley, Martin Maechler, Arni Magnusson, Steffen Moeller, Marc Schwartz and Bill Venables (2016). gplots: Various R Programming Tools for Plotting Data. R package version 3.0.1. https://CRAN.R-project.org/package=gplots |
| doParallel | 1.0.10 | Revolution Analytics and Steve Weston (2015). doParallel: Foreach Parallel Adaptor for the 'parallel' Package. R package version 1.0.10. https://CRAN.R-project.org/package=doParallel |
| iterators | 1.0.8 | Revolution Analytics and Steve Weston (2015). iterators: Provides Iterator Construct for R. R package version 1.0.8. https://CRAN.R-project.org/package=iterators |
| foreach | 1.4.3 | Revolution Analytics and Steve Weston (2015). foreach: Provides Foreach Looping Construct for R. R package version 1.4.3. https://CRAN.R-project.org/package=foreach |
| dtplyr | 0.0.1 | Hadley Wickham (2016). dtplyr: Data Table Back-End for 'dplyr'. R package version 0.0.1. https://CRAN.R-project.org/package=dtplyr |
| plyr | 1.8.4 | Hadley Wickham (2011). The Split-Apply-Combine Strategy for Data Analysis. Journal of Statistical Software, 40(1), 1-29. URL http://www.jstatsoft.org/v40/i01/ |
| dplyr | 0.5.0 | Hadley Wickham and Romain Francois (2016). dplyr: A Grammar of Data Manipulation. R package version 0.5.0. https://CRAN.R-project.org/package=dplyr |
| psych | 1.6.9 | Revelle, W. (2016) psych: Procedures for Personality and Psychological Research, Northwestern University, Evanston, Illinois, USA, https://CRAN.R-project.org/package=psych Version = 1.6.9. |
| data.table | 1.9.6 | M Dowle, A Srinivasan, T Short, S Lianoglou with contributions from R Saporta and E Antonyan (2015). data.table: Extension of Data.frame. R package version 1.9.6. https://CRAN.R-project.org/package=data.table |
| tictoc | 1.0 | Sergei Izrailev (2014). tictoc: Functions for timing R scripts, as well as implementations of Stack and List structures.. R package version 1.0. https://CRAN.R-project.org/package=tictoc |
| openxlsx | 3.0.0 | Alexander Walker (2015). openxlsx: Read, Write and Edit XLSX Files. R package version 3.0.0. https://CRAN.R-project.org/package=openxlsx |
