# Supplementary material for: Comparison of Fatty Acid and Gene Profiles in Skeletal Muscle in Normal and Obese C57BL/6J Mice before and after Blunt Muscle Injury
Source: Front Physiol. 2018 Jan 30;9:19. doi: 10.3389/fphys.2018.00019 (PMC5797686; doi:10.3389/fphys.2018.00019)
Supplement: Supplement 2.1 — Significance levels of FA-composition in phospholipid fraction in muscle tissue; n = 18 per group. Statistical analysis by two-sided homoscedastic t-test. *Indicates p ≤ 0.01, **Indicates p ≤ 0.05. [file Supplement2.1.DOCX]

Supplementary Material

Comparison of fatty acid and gene profiles in skeletal muscle in normal and obese C57BL/6J mice before and after blunt muscle injury

Jens-Uwe Werner^1†^, Klaus Tödter^2†^, Pengfei Xu^1^, Lydia Lockhart^1^, Markus Jähnert^3^, Pascal Gottmann^3^, Annette Schürmann^3^, Ludger Scheja^2^, Martin Wabitsch^4,^*, Uwe Knippschild^1,^*

* Correspondence: Prof. Dr. Martin Wabitsch, Ulm University Hospital for Pediatrics and Adolescent Medicine, Division of Pediatric Endocrinology and Diabetes, Eythstraße 24, 89075 Ulm, Germany, martin.wabitsch@uniklinik-ulm.de and Prof. Dr. Uwe Knippschild, Ulm University Hospital, Department of General and Visceral Surgery, Albert-Einstein-Allee 23, 89081 Ulm, Germany, uwe.knippschild@uniklinik-ulm.de

Supplement 2.1: Significance levels of FA-composition in phospholipid fraction in muscle tissue; n = 18 per group. Statistical analysis by two-sided homoscedastic t-test. * indicates p ≤ 0.01, ** indicates p ≤ 0.05.

|  | **Phospholipid fraction** | | | |
| --- | --- | --- | --- | --- |
|  | **Normal** | **Obese** | **Control** | **Trauma** |
|  | **Control vs Trauma** | **Control vs Trauma** | **Normal vs Obese** | **Normal vs Obese** |
| Myristic (14:0) | 5.15E-01 | 7.49E-01 | 1.74E-16* | 4.01E-05* |
| Myristoleic (14:1) | - | - | - | - |
| Palmitic (16:0) | 6.64E-01 | 7.24E-01 | 6.38E-02 | 2.22E-01 |
| d-7-hexadecenoic (16:1) | 3.08E-01 | 9.76E-01 | 7.56E-03* | 6.58E-04* |
| Palmitoleic (16:1) | 5.26E-01 | 3.31E-01 | 3.52E-22* | 4.26E-21* |
| Stearic (18:0) | 3.57E-01 | 8.31E-01 | 1.18E-04* | 5.45E-04* |
| Oleic (18:1) | 5.90E-01 | 1.40E-01 | 1.33E-02** | 4.53E-02** |
| Vaccenic (18:1) | 6.82E-01 | 6.08E-01 | 1.46E-14* | 1.84E-14* |
| Linoleic (18:2) | 1.91E-01 | 2.59E-01 | 8.15E-06* | 3.48E-03* |
| g-Linolenic (18:3) | 3.71E-01 | 3.13E-01 | 8.16E-11* | 2.94E-01 |
| Linolenic (18:3) | 9.32E-01 | 4.14E-01 | 3.15E-03* | 7.86E-01 |
| Stearidonic (18:4) | - | - | - | - |
| Arachidic (20:0) | 4.89E-01 | 6.11E-01 | 3.46E-01 | 8.25E-01 |
| Eicosenoic (20:1) | 3.86E-02** | 4.23E-01 | 4.58E-05* | 1.40E-05* |
| Eicosadienoic (20:2) | 5.46E-02 | 4.44E-01 | 1.50E-24* | 1.12E-22* |
| DHG-Linolenic (20:3) | 3.79E-01 | 2.07E-02** | 1.01E-08* | 4.57E-10* |
| Arachidonic (20:4) | 6.47E-01 | 1.65E-01 | 1.33E-03* | 3.74E-07* |
| Eicosatrienoic (20:3) | - | - | - | - |
| Eicosatetraenoic (20:4) | - | - | - | - |
| Eicosapentaenoic (20:5) | 2.78E-01 | 9.11E-01 | 1.94E-03* | 4.15E-08* |
| Behenic (22:0) | 6.32E-01 | 2.01E-01 | 2.51E-02** | 1.35E-01 |
| Erucic (22:1) | 5.05E-01 | 8.95E-01 | 5.94E-08* | 1.71E-06* |
| Docosapentaenoic (22:5) | 1.17E-01 | 3.26E-01 | 1.13E-20* | 3.67E-18* |
| Docosahexaenoic (22:6) | 2.07E-01 | 6.68E-01 | 2.28E-02** | 1.67E-04* |
| Lignoceric (24:0) | 5.88E-01 | 2.69E-01 | 7.19E-02 | 3.68E-01 |
| Nervonic (24:1) | 9.38E-01 | 1.41E-01 | 5.14E-03* | 1.38E-02** |
