# Supplementary material for: Comparison of Fatty Acid and Gene Profiles in Skeletal Muscle in Normal and Obese C57BL/6J Mice before and after Blunt Muscle Injury
Source: Front Physiol. 2018 Jan 30;9:19. doi: 10.3389/fphys.2018.00019 (PMC5797686; doi:10.3389/fphys.2018.00019)
Supplement: Supplement 2.2 — Fatty acid content in phospholipid fraction from 1 h to 21 d post-injury for female normal weight C57BL/6J mice. N.d., not detected. [file Supplement2.2.DOCX]

Supplementary Material

Comparison of fatty acid and gene profiles in skeletal muscle in normal and obese C57BL/6J mice before and after blunt muscle injury

Jens-Uwe Werner^1†^, Klaus Tödter^2†^, Pengfei Xu^1^, Lydia Lockhart^1^, Markus Jähnert^3^, Pascal Gottmann^3^, Annette Schürmann^3^, Ludger Scheja^2^, Martin Wabitsch^4,^*, Uwe Knippschild^1,^*

* Correspondence: Prof. Dr. Martin Wabitsch, Ulm University Hospital for Pediatrics and Adolescent Medicine, Division of Pediatric Endocrinology and Diabetes, Eythstraße 24, 89075 Ulm, Germany, martin.wabitsch@uniklinik-ulm.de and Prof. Dr. Uwe Knippschild, Ulm University Hospital, Department of General and Visceral Surgery, Albert-Einstein-Allee 23, 89081 Ulm, Germany, uwe.knippschild@uniklinik-ulm.de

Supplement 2.2: Fatty acid content in phospholipid fraction from 1h to 21d post-injury for female normal weight C57BL/6J mice. N.d. = not detected.

|  | **Phospholipid fraction in muscle tissue of female normal weight C57BL/6J mice** | | | | | | | | | | | | | | | | | | | | | | | |
| --- | --- | --- | --- | --- | --- | --- | --- | --- | --- | --- | --- | --- | --- | --- | --- | --- | --- | --- | --- | --- | --- | --- | --- | --- |
| **Time** | **1h** | | | | **6h** | | | | **24h** | | | | **72h** | | | | **192h** | | | | **504h** | | | |
| **Treatment** | **Control** | | **Trauma** | | **Control** | | **Trauma** | | **Control** | | **Trauma** | | **Control** | | **Trauma** | | **Control** | | **Trauma** | | **Control** | | **Trauma** | |
|  | AV | sd | AV | sd | AV | sd | AV | sd | AV | sd | AV | sd | AV | sd | AV | sd | AV | sd | AV | sd | AV | sd | AV | sd |
| Myristic (14:0) | 1.10 | 0.05 | 1.13 | 0.05 | 1.05 | 0.03 | 1.61 | 0.77 | 1.13 | 0.04 | 1.05 | 0.06 | 1.11 | 0.07 | 0.97 | 0.04 | 1.14 | 0.02 | 1.08 | 0.13 | 1.11 | 0.02 | 1.12 | 0.09 |
| Myristoleic (14:1) |  |  |  |  |  |  |  |  |  |  |  |  |  |  |  |  |  |  |  |  |  |  |  |  |
| Palmitic (16:0) | 27.99 | 2.31 | 28.92 | 0.32 | 27.64 | 0.96 | 27.42 | 0.43 | 27.96 | 0.99 | 27.60 | 0.62 | 27.69 | 0.76 | 27.93 | 1.25 | 28.00 | 0.52 | 26.12 | 2.12 | 26.89 | 1.36 | 27.12 | 0.89 |
| d-7-hexadecenoic (16:1) | 0.71 | 0.08 | 0.63 | 0.11 | 0.75 | 0.09 | 0.75 | 0.09 | 0.78 | 0.02 | 0.79 | 0.02 | 0.84 | 0.25 | 0.82 | 0.09 | 0.75 | 0.13 | 0.91 | 0.11 | 0.78 | 0.01 | 0.94 | 0.06 |
| Palmitoleic (16:1) | 1.92 | 0.16 | 2.15 | 0.08 | 1.95 | 0.11 | 2.14 | 0.05 | 2.21 | 0.09 | 2.12 | 0.26 | 2.09 | 0.14 | 1.77 | 0.27 | 2.07 | 0.32 | 2.32 | 0.24 | 2.29 | 0.39 | 2.36 | 0.18 |
| Stearic (18:0) | 16.22 | 2.62 | 17.08 | 0.54 | 18.08 | 0.77 | 16.47 | 1.16 | 16.29 | 2.31 | 15.97 | 0.55 | 15.64 | 0.69 | 17.37 | 0.75 | 16.37 | 2.51 | 17.56 | 0.35 | 14.99 | 0.29 | 15.82 | 0.66 |
| Oleic (18:1) | 5.73 | 0.20 | 5.68 | 0.31 | 5.65 | 0.38 | 6.41 | 0.50 | 6.91 | 1.38 | 5.72 | 0.24 | 6.14 | 0.73 | 6.87 | 0.81 | 6.43 | 1.78 | 9.69 | 2.62 | 8.24 | 4.11 | 6.68 | 0.53 |
| Vaccenic (18:1) | 4.57 | 0.27 | 4.06 | 0.30 | 4.17 | 0.22 | 4.34 | 0.35 | 4.33 | 0.53 | 4.58 | 0.12 | 4.63 | 0.04 | 4.41 | 0.28 | 4.51 | 0.38 | 4.31 | 0.47 | 4.63 | 0.17 | 4.85 | 0.22 |
| Linoleic (18:2) | 7.63 | 0.75 | 8.18 | 0.93 | 7.60 | 0.42 | 7.66 | 0.50 | 7.42 | 0.54 | 7.46 | 0.40 | 8.05 | 0.69 | 7.75 | 1.41 | 7.22 | 1.05 | 7.52 | 1.00 | 6.96 | 0.78 | 8.53 | 1.37 |
| g-Linolenic (18:3) | 0.08 | 0.02 | 0.09 | 0.01 | 0.08 | 0.01 | 0.09 | 0.01 | 0.08 | 0.02 | 0.09 | 0.01 | 0.08 | 0.00 | 0.08 | 0.02 | 0.08 | 0.00 | 0.08 | 0.00 | 0.08 | 0.01 | 0.08 | 0.00 |
| Linolenic (18:3) | 0.60 | 0.07 | 0.65 | 0.02 | 0.52 | 0.01 | 0.55 | 0.01 | 0.55 | 0.05 | 0.58 | 0.01 | 0.57 | 0.02 | 0.53 | 0.03 | 0.50 | 0.03 | 0.47 | 0.03 | 0.53 | 0.03 | 0.51 | 0.01 |
| Stearidonic (18:4) |  |  |  |  |  |  |  |  |  |  |  |  |  |  |  |  |  |  |  |  |  |  |  |  |
| Arachidic (20:0) | 0.23 | 0.06 | 0.18 | 0.02 | 0.17 | 0.00 | 0.19 | 0.05 | 0.25 | 0.09 | 0.16 | 0.02 | 0.18 | 0.08 | 0.21 | 0.07 | 0.24 | 0.18 | 0.31 | 0.24 | 0.31 | 0.26 | 0.16 | 0.03 |
| Eicosenoic (20:1) | 0.16 | 0.04 | 0.16 | 0.01 | 0.16 | 0.01 | 0.20 | 0.02 | 0.20 | 0.01 | 0.19 | 0.03 | 0.18 | 0.01 | 0.22 | 0.04 | 0.17 | 0.02 | 0.22 | 0.02 | 0.18 | 0.02 | 0.18 | 0.00 |
| Eicosadienoic (20:2) | 0.26 | 0.06 | 0.35 | 0.06 | 0.29 | 0.03 | 0.30 | 0.04 | 0.29 | 0.05 | 0.33 | 0.01 | 0.30 | 0.03 | 0.38 | 0.09 | 0.28 | 0.01 | 0.27 | 0.01 | 0.27 | 0.03 | 0.27 | 0.04 |
| DHG-Linolenic (20:3) | 0.94 | 0.05 | 0.86 | 0.10 | 0.91 | 0.07 | 0.94 | 0.04 | 0.95 | 0.05 | 1.00 | 0.02 | 0.95 | 0.06 | 0.88 | 0.03 | 0.91 | 0.10 | 0.80 | 0.03 | 0.89 | 0.09 | 0.94 | 0.03 |
| Arachidonic (20:4) | 12.09 | 0.61 | 11.66 | 0.50 | 11.46 | 0.81 | 11.47 | 0.32 | 11.78 | 0.76 | 12.68 | 0.86 | 12.35 | 0.57 | 12.05 | 0.64 | 12.57 | 1.85 | 11.03 | 1.09 | 11.61 | 0.71 | 12.17 | 0.39 |
| Eicosatrienoic (20:3) |  |  |  |  |  |  |  |  |  |  |  |  |  |  |  |  |  |  |  |  |  |  |  |  |
| Eicosatetraenoic (20:4) |  |  |  |  |  |  |  |  |  |  |  |  |  |  |  |  |  |  |  |  |  |  |  |  |
| Eicosapentaenoic (20:5) | 0.05 | 0.05 | 0.08 | 0.01 | 0.07 | 0.00 | 0.08 | 0.01 | 0.09 | 0.01 | 0.08 | 0.00 | 0.08 | 0.01 | 0.09 | 0.00 | 0.08 | 0.01 | 0.08 | 0.01 | 0.08 | 0.02 | 0.09 | 0.00 |
| Behenic (22:0) | 0.21 | 0.05 | 0.25 | 0.05 | 0.23 | 0.04 | 0.31 | 0.12 | 0.39 | 0.15 | 0.22 | 0.01 | 0.29 | 0.10 | 0.32 | 0.10 | 0.36 | 0.34 | 0.51 | 0.53 | 0.59 | 0.67 | 0.21 | 0.02 |
| Erucic (22:1) | 0.09 | 0.03 | 0.09 | 0.00 | 0.08 | 0.00 | 0.10 | 0.01 | 0.10 | 0.01 | 0.12 | 0.06 | 0.10 | 0.02 | 0.10 | 0.01 | 0.09 | 0.02 | 0.10 | 0.03 | 0.09 | 0.01 | 0.08 | 0.01 |
| Docosapentaenoic (22:5) | 1.21 | 0.25 | 1.26 | 0.08 | 1.17 | 0.04 | 1.27 | 0.07 | 1.18 | 0.21 | 1.34 | 0.09 | 1.12 | 0.05 | 1.30 | 0.03 | 1.11 | 0.03 | 1.02 | 0.06 | 1.07 | 0.12 | 1.10 | 0.07 |
| Docosahexaenoic (22:6) | 17.56 | 3.61 | 15.87 | 1.30 | 17.32 | 1.69 | 16.77 | 1.39 | 15.89 | 2.69 | 17.26 | 1.84 | 16.74 | 1.65 | 14.75 | 1.74 | 15.94 | 2.24 | 13.82 | 1.47 | 16.54 | 4.09 | 16.06 | 0.97 |
| Lignoceric (24:0) | 0.29 | 0.02 | 0.30 | 0.07 | 0.26 | 0.09 | 0.40 | 0.17 | 0.56 | 0.24 | 0.22 | 0.02 | 0.37 | 0.17 | 0.45 | 0.21 | 0.55 | 0.59 | 0.83 | 0.89 | 0.96 | 1.23 | 0.28 | 0.08 |
| Nervonic (24:1) | 0.36 | 0.04 | 0.38 | 0.05 | 0.38 | 0.08 | 0.47 | 0.14 | 0.56 | 0.16 | 0.36 | 0.02 | 0.40 | 0.11 | 0.65 | 0.21 | 0.53 | 0.40 | 0.84 | 0.64 | 0.78 | 0.77 | 0.36 | 0.01 |
