# Supplementary material for: Comparison of Fatty Acid and Gene Profiles in Skeletal Muscle in Normal and Obese C57BL/6J Mice before and after Blunt Muscle Injury
Source: Front Physiol. 2018 Jan 30;9:19. doi: 10.3389/fphys.2018.00019 (PMC5797686; doi:10.3389/fphys.2018.00019)
Supplement: Supplement 2.3 — Fatty acid content in phospholipid fraction from 1 h to 21 d post-injury for female obese C57BL/6J mice. N.d., not detected. [file Supplement2.3.DOCX]

Supplementary Material

Comparison of fatty acid and gene profiles in skeletal muscle in normal and obese C57BL/6J mice before and after blunt muscle injury

Jens-Uwe Werner^1†^, Klaus Tödter^2†^, Pengfei Xu^1^, Lydia Lockhart^1^, Markus Jähnert^3^, Pascal Gottmann^3^, Annette Schürmann^3^, Ludger Scheja^2^, Martin Wabitsch^4,^*, Uwe Knippschild^1,^*

* Correspondence: Prof. Dr. Martin Wabitsch, Ulm University Hospital for Pediatrics and Adolescent Medicine, Division of Pediatric Endocrinology and Diabetes, Eythstraße 24, 89075 Ulm, Germany, martin.wabitsch@uniklinik-ulm.de and Prof. Dr. Uwe Knippschild, Ulm University Hospital, Department of General and Visceral Surgery, Albert-Einstein-Allee 23, 89081 Ulm, Germany, uwe.knippschild@uniklinik-ulm.de

Supplement 2.3: Fatty acid content in phospholipid fraction from 1h to 21d post-injury for female obese C57BL/6J mice. N.d. = not detected.

|  | **Phospholipid fraction in muscle tissue of female obese C57BL/6J mice** | | | | | | | | | | | | | | | | | | | | | | | |
| --- | --- | --- | --- | --- | --- | --- | --- | --- | --- | --- | --- | --- | --- | --- | --- | --- | --- | --- | --- | --- | --- | --- | --- | --- |
| **Time** | **1h** | | | | **6h** | | | | **24h** | | | | **72h** | | | | **192h** | | | | **504h** | | | |
| **Treatment** | **Control** | | **Trauma** | | **Control** | | **Trauma** | | **Control** | | **Trauma** | | **Control** | | **Trauma** | | **Control** | | **Trauma** | | **Control** | | **Trauma** | |
|  | AV | sd | AV | sd | AV | sd | AV | sd | AV | sd | AV | sd | AV | sd | AV | sd | AV | sd | AV | sd | AV | sd | AV | sd |
| Myristic (14:0) | 0.76 | 0.08 | 0.82 | 0.04 | 0.85 | 0.11 | 0.73 | 0.03 | 0.75 | 0.07 | 0.72 | 0.02 | 0.71 | 0.06 | 0.74 | 0.06 | 0.76 | 0.04 | 0.80 | 0.05 | 0.84 | 0.07 | 0.82 | 0.02 |
| Myristoleic (14:1) | n.d. |  | n.d. |  | n.d. |  | n.d. |  | n.d. |  | n.d. |  | n.d. |  | n.d. |  | n.d. |  | n.d. |  | n.d. |  | n.d. |  |
| Palmitic (16:0) | 26.61 | 1.12 | 28.05 | 1.62 | 28.11 | 2.88 | 26.24 | 1.39 | 26.15 | 1.37 | 27.47 | 1.67 | 26.22 | 1.41 | 27.35 | 2.05 | 26.90 | 1.39 | 26.34 | 0.32 | 26.69 | 2.15 | 26.31 | 0.83 |
| d-7-hexadecenoic (16:1) | 0.71 | 0.14 | 0.69 | 0.09 | 0.70 | 0.14 | 0.67 | 0.03 | 0.60 | 0.01 | 0.58 | 0.08 | 0.66 | 0.01 | 0.62 | 0.03 | 0.67 | 0.14 | 0.79 | 0.12 | 0.67 | 0.04 | 0.67 | 0.04 |
| Palmitoleic (16:1) | 0.61 | 0.07 | 0.69 | 0.10 | 0.69 | 0.09 | 0.66 | 0.12 | 0.65 | 0.17 | 0.58 | 0.07 | 0.69 | 0.04 | 0.81 | 0.07 | 0.70 | 0.13 | 0.82 | 0.07 | 0.83 | 0.05 | 0.83 | 0.02 |
| Stearic (18:0) | 18.94 | 1.61 | 17.63 | 4.19 | 17.67 | 2.40 | 19.44 | 0.59 | 18.47 | 1.76 | 20.94 | 2.91 | 18.95 | 1.07 | 19.00 | 1.21 | 20.16 | 1.32 | 18.59 | 0.46 | 18.05 | 0.71 | 17.46 | 0.40 |
| Oleic (18:1) | 4.97 | 0.22 | 4.86 | 0.38 | 4.81 | 0.74 | 5.51 | 0.21 | 4.91 | 0.30 | 4.61 | 0.20 | 4.96 | 0.42 | 5.13 | 0.34 | 5.35 | 1.08 | 6.90 | 0.55 | 6.57 | 0.59 | 7.79 | 1.05 |
| Vaccenic (18:1) | 3.07 | 0.22 | 3.31 | 0.20 | 3.45 | 0.44 | 3.08 | 0.03 | 3.30 | 0.33 | 3.00 | 0.20 | 3.22 | 0.06 | 3.31 | 0.01 | 3.18 | 0.15 | 3.33 | 0.15 | 3.34 | 0.17 | 3.31 | 0.11 |
| Linoleic (18:2) | 9.78 | 0.91 | 7.86 | 0.12 | 8.62 | 2.77 | 9.70 | 0.60 | 9.83 | 0.48 | 8.41 | 0.92 | 9.15 | 1.30 | 8.33 | 1.63 | 9.17 | 1.21 | 9.84 | 0.38 | 9.75 | 1.48 | 9.34 | 0.96 |
| g-Linolenic (18:3) | 0.06 | 0.00 | 0.14 | 0.14 | 0.06 | 0.01 | 0.06 | 0.00 | 0.05 | 0.00 | 0.05 | 0.01 | 0.06 | 0.00 | 0.06 | 0.00 | 0.05 | 0.01 | 0.06 | 0.00 | 0.06 | 0.00 | 0.06 | 0.00 |
| Linolenic (18:3) | 0.35 | 0.02 | 1.85 | 2.53 | 0.61 | 0.43 | 0.35 | 0.01 | 0.37 | 0.05 | 0.34 | 0.03 | 0.37 | 0.01 | 0.37 | 0.00 | 0.35 | 0.03 | 0.38 | 0.02 | 0.42 | 0.03 | 0.41 | 0.00 |
| Stearidonic (18:4) | n.d. |  | n.d. |  | n.d. |  | n.d. |  | n.d. |  | n.d. |  | n.d. |  | n.d. |  | n.d. |  | n.d. |  | n.d. |  | n.d. |  |
| Arachidic (20:0) | 0.17 | 0.01 | 0.33 | 0.23 | 0.32 | 0.26 | 0.17 | 0.01 | 0.15 | 0.01 | 0.18 | 0.04 | 0.16 | 0.02 | 0.16 | 0.02 | 0.19 | 0.01 | 0.18 | 0.02 | 0.16 | 0.02 | 0.24 | 0.11 |
| Eicosenoic (20:1) | 0.15 | 0.01 | 0.14 | 0.01 | 0.14 | 0.02 | 0.16 | 0.01 | 0.14 | 0.01 | 0.13 | 0.01 | 0.14 | 0.01 | 0.13 | 0.02 | 0.14 | 0.03 | 0.17 | 0.01 | 0.17 | 0.02 | 0.18 | 0.00 |
| Eicosadienoic (20:2) | 0.80 | 0.04 | 0.85 | 0.06 | 0.89 | 0.10 | 0.81 | 0.04 | 0.83 | 0.09 | 0.76 | 0.11 | 0.79 | 0.03 | 0.75 | 0.03 | 0.72 | 0.03 | 0.74 | 0.03 | 0.79 | 0.06 | 0.80 | 0.02 |
| DHG-Linolenic (20:3) | 0.80 | 0.07 | 0.72 | 0.01 | 0.75 | 0.08 | 0.72 | 0.05 | 0.81 | 0.01 | 0.70 | 0.09 | 0.76 | 0.07 | 0.70 | 0.07 | 0.74 | 0.06 | 0.75 | 0.04 | 0.72 | 0.06 | 0.70 | 0.03 |
| Arachidonic (20:4) | 10.53 | 0.60 | 10.24 | 0.65 | 11.81 | 1.82 | 9.70 | 0.37 | 11.61 | 1.70 | 10.00 | 0.64 | 10.23 | 0.12 | 11.36 | 0.22 | 10.14 | 0.28 | 10.33 | 0.46 | 10.21 | 0.67 | 10.22 | 0.38 |
| Eicosatrienoic (20:3) | n.d. |  | n.d. |  | n.d. |  | n.d. |  | n.d. |  | n.d. |  | n.d. |  | n.d. |  | n.d. |  | n.d. |  | n.d. |  | n.d. |  |
| Eicosatetraenoic (20:4) | n.d. |  | n.d. |  | n.d. |  | n.d. |  | n.d. |  | n.d. |  | n.d. |  | n.d. |  | n.d. |  | n.d. |  | n.d. |  | n.d. |  |
| Eicosapentaenoic (20:5) | 0.06 | 0.01 | 0.04 | 0.03 | 0.03 | 0.03 | 0.06 | 0.00 | 0.06 | 0.00 | 0.06 | 0.00 | 0.06 | 0.00 | 0.06 | 0.00 | 0.06 | 0.01 | 0.06 | 0.00 | 0.06 | 0.00 | 0.06 | 0.00 |
| Behenic (22:0) | 0.21 | 0.02 | 0.18 | 0.04 | 0.16 | 0.04 | 0.20 | 0.01 | 0.17 | 0.02 | 0.17 | 0.02 | 0.20 | 0.02 | 0.19 | 0.02 | 0.18 | 0.01 | 0.20 | 0.03 | 0.17 | 0.02 | 0.36 | 0.25 |
| Erucic (22:1) | 0.07 | 0.01 | 0.07 | 0.01 | 0.08 | 0.02 | 0.07 | 0.01 | 0.05 | 0.02 | 0.04 | 0.01 | 0.05 | 0.01 | 0.04 | 0.01 | 0.04 | 0.01 | 0.04 | 0.01 | 0.05 | 0.00 | 0.07 | 0.01 |
| Docosapentaenoic (22:5) | 2.13 | 0.20 | 2.21 | 0.13 | 2.18 | 0.24 | 1.99 | 0.12 | 2.28 | 0.22 | 2.14 | 0.23 | 2.18 | 0.08 | 2.30 | 0.06 | 2.12 | 0.12 | 1.98 | 0.16 | 2.02 | 0.06 | 1.95 | 0.07 |
| Docosahexaenoic (22:6) | 18.47 | 0.92 | 18.69 | 1.73 | 17.45 | 0.88 | 18.94 | 1.29 | 18.15 | 2.15 | 18.50 | 2.00 | 19.75 | 0.68 | 17.88 | 1.94 | 17.71 | 0.49 | 16.93 | 0.17 | 17.80 | 1.34 | 17.19 | 1.02 |
| Lignoceric (24:0) | 0.30 | 0.04 | 0.27 | 0.04 | 0.28 | 0.11 | 0.27 | 0.04 | 0.24 | 0.06 | 0.22 | 0.03 | 0.25 | 0.04 | 0.25 | 0.05 | 0.26 | 0.04 | 0.31 | 0.07 | 0.24 | 0.04 | 0.60 | 0.46 |
| Nervonic (24:1) | 0.28 | 0.04 | 0.23 | 0.05 | 0.22 | 0.06 | 0.28 | 0.04 | 0.26 | 0.04 | 0.25 | 0.03 | 0.29 | 0.03 | 0.30 | 0.03 | 0.26 | 0.02 | 0.30 | 0.05 | 0.24 | 0.04 | 0.48 | 0.29 |
