# Supplementary material for: Comparison of Fatty Acid and Gene Profiles in Skeletal Muscle in Normal and Obese C57BL/6J Mice before and after Blunt Muscle Injury
Source: Front Physiol. 2018 Jan 30;9:19. doi: 10.3389/fphys.2018.00019 (PMC5797686; doi:10.3389/fphys.2018.00019)
Supplement: Supplement 2.4 — Significance levels of FA-composition in triglyceride fraction in muscle tissue; n = 18 per group. Statistical analysis by two-sided homoscedastic t-test. *Indicates p ≤ 0.01, **Indicates p ≤ 0.05. [file Supplement2.4.DOCX]

Supplementary Material

Comparison of fatty acid and gene profiles in skeletal muscle in normal and obese C57BL/6J mice before and after blunt muscle injury

Jens-Uwe Werner^1†^, Klaus Tödter^2†^, Pengfei Xu^1^, Lydia Lockhart^1^, Markus Jähnert^3^, Pascal Gottmann^3^, Annette Schürmann^3^, Ludger Scheja^2^, Martin Wabitsch^4,^*, Uwe Knippschild^1,^*

* Correspondence: Prof. Dr. Martin Wabitsch, Ulm University Hospital for Pediatrics and Adolescent Medicine, Division of Pediatric Endocrinology and Diabetes, Eythstraße 24, 89075 Ulm, Germany, martin.wabitsch@uniklinik-ulm.de and Prof. Dr. Uwe Knippschild, Ulm University Hospital, Department of General and Visceral Surgery, Albert-Einstein-Allee 23, 89081 Ulm, Germany, uwe.knippschild@uniklinik-ulm.de

Supplement 2.4: Significance levels of FA-composition in triglyceride fraction in muscle tissue; n = 18 per group. Statistical analysis by two-sided homoscedastic t-test. * indicates p ≤ 0.01, ** indicates p ≤ 0.05.

|  | **Triglyceride fraction** | | | |
| --- | --- | --- | --- | --- |
|  | **Normal** | **Obese** | **Control** | **Trauma** |
|  | **Control vs Trauma** | **Control vs Trauma** | **Normal vs Obese** | **Normal vs Obese** |
| Myristic (14:0) | 3.43E-01 | 2.85E-01 | 3.98E-18* | 3.05E-21* |
| Myristoleic (14:1) | 9.06E-01 | 7.62E-01 | 6.88E-11* | 5.29E-12* |
| Palmitic (16:0) | 4.42E-01 | 4.31E-01 | 5.04E-10* | 7.12E-13* |
| d-7-hexadecenoic (16:1) | 6.58E-02 | 1.01E-01 | 2.51E-02** | 1.86E-01 |
| Palmitoleic (16:1) | 9.76E-01 | 9.22E-01 | 1.85E-10* | 3.18E-09* |
| Stearic (18:0) | 3.20E-01 | 5.82E-01 | 4.98E-02** | 1.12E-01 |
| Oleic (18:1) | 1.61E-01 | 4.57E-01 | 1.77E-03* | 2.15E-03* |
| Vaccenic (18:1) | 1.60E-01 | 2.72E-01 | 1.20E-01 | 5.97E-02 |
| Linoleic (18:2) | 5.70E-01 | 5.76E-01 | 5.81E-18* | 5.12E-19* |
| g-Linolenic (18:3) | 2.36E-01 | 3.99E-01 | 1.39E-05* | 9.32E-07* |
| Linolenic (18:3) | 3.37E-01 | 1.70E-01 | 8.89E-04* | 7.02E-12* |
| Stearidonic (18:4) | - | - | - | - |
| Arachidic (20:0) | 5.39E-01 | 5.42E-01 | 8.69E-03* | 1.79E-06* |
| Eicosenoic (20:1) | 4.15E-01 | 6.44E-01 | 2.13E-03* | 9.70E-05* |
| Eicosadienoic (20:2) | 4.22E-01 | 2.12E-01 | 8.58E-27* | 6.91E-25* |
| DHG-Linolenic (20:3) | 4.68E-01 | 6.82E-01 | 2.75E-09* | 2.39E-08* |
| Arachidonic (20:4) | 2.11E-01 | 7.06E-01 | 3.58E-09* | 2.24E-06* |
| Eicosatrienoic (20:3) | - | - | - | - |
| Eicosatetraenoic (20:4) | - | - | - | - |
| Eicosapentaenoic (20:5) | 3.45E-01 | 1.14E-01 | 1.34E-09* | 1.34E-06* |
| Behenic (22:0) | 9.03E-01 | 6.72E-01 | 3.04E-04* | 2.47E-03* |
| Erucic (22:1) | 7.93E-01 | 8.18E-01 | 1.60E-09* | 4.69E-12* |
| Docosapentaenoic (22:5) | 5.53E-01 | 2.60E-01 | 1.19E-06* | 1.15E-04* |
| Docosahexaenoic (22:6) | 1.49E-02** | 7.31E-01 | 2.40E-10* | 4.27E-04* |
| Lignoceric (24:0) | 9.90E-01 | 9.78E-01 | 2.93E-03* | 9.03E-03* |
| Nervonic (24:1) | 3.41E-01 | 7.08E-01 | 3.11E-02** | 6.83E-01 |
