# Supplementary material for: Comparison of Fatty Acid and Gene Profiles in Skeletal Muscle in Normal and Obese C57BL/6J Mice before and after Blunt Muscle Injury
Source: Front Physiol. 2018 Jan 30;9:19. doi: 10.3389/fphys.2018.00019 (PMC5797686; doi:10.3389/fphys.2018.00019)
Supplement: Supplement 2.5 — Fatty acid content in triglyceride fraction from 1h to 21d post-injury for female normal weight C57BL/6J mice. N.d., not detected. [file Supplement2.5.DOCX]

Supplementary Material

Comparison of fatty acid and gene profiles in skeletal muscle in normal and obese C57BL/6J mice before and after blunt muscle injury

Jens-Uwe Werner^1†^, Klaus Tödter^2†^, Pengfei Xu^1^, Lydia Lockhart^1^, Markus Jähnert^3^, Pascal Gottmann^3^, Annette Schürmann^3^, Ludger Scheja^2^, Martin Wabitsch^4,^*, Uwe Knippschild^1,^*

* Correspondence: Prof. Dr. Martin Wabitsch, Ulm University Hospital for Pediatrics and Adolescent Medicine, Division of Pediatric Endocrinology and Diabetes, Eythstraße 24, 89075 Ulm, Germany, martin.wabitsch@uniklinik-ulm.de and Prof. Dr. Uwe Knippschild, Ulm University Hospital, Department of General and Visceral Surgery, Albert-Einstein-Allee 23, 89081 Ulm, Germany, uwe.knippschild@uniklinik-ulm.de

Supplement 2.5: Fatty acid content in triglyceride fraction from 1h to 21d post-injury for female normal weight C57BL/6J mice. N.d. = not detected.

|  | **Trigylceride fraction in muscle tissue of female normal weight C57BL/6J mice** | | | | | | | | | | | | | | | | | | | | | | | |
| --- | --- | --- | --- | --- | --- | --- | --- | --- | --- | --- | --- | --- | --- | --- | --- | --- | --- | --- | --- | --- | --- | --- | --- | --- |
| **Time** | **1h** | | | | **6h** | | | | **24h** | | | | **72h** | | | | **192h** | | | | **504h** | | | |
| **Treatment** | **Control** | | **Trauma** | | **Control** | | **Trauma** | | **Control** | | **Trauma** | | **Control** | | **Trauma** | | **Control** | | **Trauma** | | **Control** | | **Trauma** | |
|  | AV | sd | AV | sd | AV | sd | AV | sd | AV | sd | AV | sd | AV | sd | AV | sd | AV | sd | AV | sd | AV | sd | AV | sd |
| Myristic (14:0) | 2.05 | 0.12 | 2.20 | 0.15 | 2.28 | 0.38 | 2.09 | 0.18 | 2.03 | 0.13 | 2.00 | 0.10 | 2.09 | 0.22 | 1.89 | 0.19 | 2.09 | 0.08 | 1.97 | 0.18 | 2.01 | 0.15 | 2.04 | 0.09 |
| Myristoleic (14:1) | 0.11 | 0.04 | 0.16 | 0.01 | 0.11 | 0.04 | 0.13 | 0.03 | 0.17 | 0.01 | 0.15 | 0.01 | 0.18 | 0.01 | 0.13 | 0.06 | 0.23 | 0.02 | 0.21 | 0.06 | 0.18 | 0.03 | 0.21 | 0.04 |
| Palmitic (16:0) | 27.06 | 3.48 | 27.59 | 1.58 | 28.19 | 4.72 | 25.42 | 2.03 | 24.77 | 1.54 | 25.64 | 1.69 | 26.45 | 3.23 | 24.37 | 1.10 | 25.64 | 0.08 | 25.17 | 2.84 | 25.10 | 1.63 | 25.44 | 0.92 |
| d-7-hexadecenoic (16:1) | 0.83 | 0.12 | 0.63 | 0.15 | 0.87 | 0.05 | 0.75 | 0.10 | 0.79 | 0.01 | 0.76 | 0.05 | 0.71 | 0.05 | 0.79 | 0.09 | 0.86 | 0.06 | 0.85 | 0.11 | 0.83 | 0.03 | 0.75 | 0.14 |
| Palmitoleic (16:1) | 6.87 | 1.57 | 8.26 | 0.47 | 6.68 | 1.09 | 7.04 | 1.29 | 8.36 | 0.35 | 7.71 | 0.82 | 9.05 | 0.75 | 6.27 | 2.25 | 10.14 | 0.63 | 10.01 | 1.84 | 9.08 | 2.11 | 10.98 | 2.04 |
| Stearic (18:0) | 9.40 | 6.69 | 7.38 | 0.90 | 11.58 | 8.07 | 7.30 | 2.06 | 7.36 | 3.08 | 7.16 | 1.75 | 6.15 | 2.18 | 8.34 | 0.45 | 8.25 | 0.51 | 7.04 | 2.30 | 6.33 | 3.04 | 5.07 | 1.78 |
| Oleic (18:1) | 37.66 | 4.79 | 37.88 | 1.44 | 35.29 | 9.15 | 40.79 | 2.82 | 38.59 | 2.54 | 39.48 | 2.78 | 37.75 | 3.31 | 39.10 | 1.59 | 36.31 | 0.41 | 37.48 | 3.67 | 38.87 | 2.41 | 39.25 | 0.48 |
| Vaccenic (18:1) | 3.29 | 0.55 | 2.88 | 0.63 | 3.16 | 0.73 | 3.08 | 0.20 | 3.13 | 1.02 | 3.16 | 0.40 | 3.16 | 0.15 | 3.16 | 0.34 | 3.50 | 0.07 | 3.10 | 0.41 | 3.64 | 0.39 | 3.21 | 0.24 |
| Linoleic (18:2) | 10.33 | 3.41 | 10.85 | 1.67 | 9.34 | 3.44 | 10.90 | 2.07 | 12.41 | 1.20 | 11.15 | 1.16 | 11.92 | 1.31 | 13.04 | 1.61 | 10.53 | 0.79 | 11.51 | 3.05 | 11.39 | 0.46 | 10.68 | 1.36 |
| g-Linolenic (18:3) | 0.08 | 0.02 | 0.07 | 0.01 | 0.10 | 0.05 | 0.07 | 0.02 | 0.07 | 0.01 | 0.07 | 0.01 | 0.07 | 0.00 | 0.07 | 0.00 | 0.07 | 0.01 | 0.08 | 0.01 | 0.07 | 0.01 | 0.07 | 0.01 |
| Linolenic (18:3) | 0.61 | 0.04 | 0.66 | 0.06 | 0.92 | 0.70 | 0.61 | 0.06 | 0.73 | 0.06 | 0.63 | 0.03 | 0.70 | 0.03 | 0.65 | 0.07 | 0.60 | 0.09 | 0.67 | 0.13 | 0.67 | 0.09 | 0.62 | 0.13 |
| Stearidonic (18:4) | n.d. |  | n.d. |  | n.d. |  | n.d. |  | n.d. |  | n.d. |  | n.d. |  | n.d. |  | n.d. |  | n.d. |  | n.d. |  | n.d. |  |
| Arachidic (20:0) | 0.24 | 0.07 | 0.19 | 0.07 | 0.25 | 0.17 | 0.16 | 0.02 | 0.14 | 0.01 | 0.19 | 0.02 | 0.14 | 0.02 | 0.17 | 0.02 | 0.15 | 0.01 | 0.14 | 0.01 | 0.13 | 0.04 | 0.11 | 0.04 |
| Eicosenoic (20:1) | 0.70 | 0.03 | 0.61 | 0.08 | 0.70 | 0.03 | 0.75 | 0.13 | 0.70 | 0.06 | 0.72 | 0.08 | 0.59 | 0.06 | 0.77 | 0.18 | 0.51 | 0.06 | 0.59 | 0.18 | 0.63 | 0.05 | 0.56 | 0.07 |
| Eicosadienoic (20:2) | 0.18 | 0.01 | 0.21 | 0.04 | 0.20 | 0.02 | 0.20 | 0.04 | 0.21 | 0.04 | 0.22 | 0.03 | 0.21 | 0.01 | 0.24 | 0.04 | 0.18 | 0.01 | 0.18 | 0.03 | 0.19 | 0.02 | 0.16 | 0.01 |
| DHG-Linolenic (20:3) | 0.12 | 0.02 | 0.11 | 0.03 | 0.14 | 0.02 | 0.12 | 0.03 | 0.13 | 0.03 | 0.14 | 0.02 | 0.13 | 0.02 | 0.13 | 0.01 | 0.12 | 0.00 | 0.13 | 0.01 | 0.13 | 0.01 | 0.11 | 0.02 |
| Arachidonic (20:4) | 0.28 | 0.04 | 0.27 | 0.05 | 0.23 | 0.06 | 0.25 | 0.04 | 0.28 | 0.04 | 0.32 | 0.02 | 0.32 | 0.04 | 0.38 | 0.04 | 0.37 | 0.03 | 0.41 | 0.04 | 0.34 | 0.03 | 0.36 | 0.11 |
| Eicosatrienoic (20:3) | n.d. |  | n.d. |  | n.d. |  | n.d. |  | n.d. |  | n.d. |  | n.d. |  | n.d. |  | n.d. |  | n.d. |  | n.d. |  | n.d. |  |
| Eicosatetraenoic (20:4) | n.d. |  | n.d. |  | n.d. |  | n.d. |  | n.d. |  | n.d. |  | n.d. |  | n.d. |  | n.d. |  | n.d. |  | n.d. |  | n.d. |  |
| Eicosapentaenoic (20:5) | n.d. |  | n.d. |  | n.d. |  | 0.02 | 0.01 | 0.02 | 0.00 | 0.02 | 0.00 | 0.02 | 0.01 | 0.02 | 0.00 | 0.02 | 0.00 | 0.02 | 0.00 | 0.02 | 0.00 | 0.01 | 0.01 |
| Behenic (22:0) | 0.06 |  | n.d. |  | n.d. |  | 0.06 | 0.03 | 0.04 | 0.01 | 0.06 | 0.01 | 0.05 | 0.01 | 0.05 | 0.01 | 0.05 | 0.00 | 0.04 | 0.00 | 0.04 | 0.02 | 0.04 | 0.01 |
| Erucic (22:1) | 0.07 | 0.03 | 0.07 | 0.02 | 0.07 | 0.01 | 0.06 | 0.02 | 0.06 | 0.02 | 0.07 | 0.01 | 0.06 | 0.01 | 0.07 | 0.01 | 0.07 | 0.01 | 0.05 | 0.00 | 0.06 | 0.04 | 0.06 | 0.01 |
| Docosapentaenoic (22:5) | 0.05 |  | n.d. |  | n.d. |  | 0.08 | 0.05 | 0.06 | 0.02 | 0.08 | 0.03 | 0.07 | 0.01 | 0.07 | 0.00 | 0.08 | 0.00 | 0.07 | 0.02 | 0.07 | 0.04 | 0.06 | 0.00 |
| Docosahexaenoic (22:6) | 0.16 |  | n.d. |  | n.d. |  | 0.15 | 0.07 | 0.14 | 0.03 | 0.20 | 0.03 | 0.14 | 0.01 | 0.22 | 0.05 | 0.17 | 0.01 | 0.20 | 0.03 | 0.16 | 0.02 | 0.17 | 0.05 |
| Lignoceric (24:0) | 0.04 |  | n.d. |  | n.d. |  | 0.05 | 0.03 | 0.03 | 0.01 | 0.04 | 0.02 | 0.03 | 0.01 | 0.03 | 0.01 | 0.05 | 0.01 | 0.03 | 0.01 | 0.03 | 0.02 | 0.02 | 0.01 |
| Nervonic (24:1) | 0.06 |  | n.d. |  | n.d. |  | 0.05 | 0.03 | 0.04 | 0.02 | 0.04 | 0.02 | 0.04 | 0.02 | 0.03 | 0.01 | 0.03 | 0.01 | 0.03 | 0.00 | 0.03 | 0.01 | 0.02 | 0.00 |
