# Supplementary material for: Comparison of Fatty Acid and Gene Profiles in Skeletal Muscle in Normal and Obese C57BL/6J Mice before and after Blunt Muscle Injury
Source: Front Physiol. 2018 Jan 30;9:19. doi: 10.3389/fphys.2018.00019 (PMC5797686; doi:10.3389/fphys.2018.00019)
Supplement: Supplement 2.6 — Fatty acid content in triglyceride fraction from 1h to 21d post-injury for female obese C57BL/6J mice. N.d., not detected. [file Supplement2.6.DOCX]

Supplementary Material

Comparison of fatty acid and gene profiles in skeletal muscle in normal and obese C57BL/6J mice before and after blunt muscle injury

Jens-Uwe Werner^1†^, Klaus Tödter^2†^, Pengfei Xu^1^, Lydia Lockhart^1^, Markus Jähnert^3^, Pascal Gottmann^3^, Annette Schürmann^3^, Ludger Scheja^2^, Martin Wabitsch^4,^*, Uwe Knippschild^1,^*

* Correspondence: Prof. Dr. Martin Wabitsch, Ulm University Hospital for Pediatrics and Adolescent Medicine, Division of Pediatric Endocrinology and Diabetes, Eythstraße 24, 89075 Ulm, Germany, martin.wabitsch@uniklinik-ulm.de and Prof. Dr. Uwe Knippschild, Ulm University Hospital, Department of General and Visceral Surgery, Albert-Einstein-Allee 23, 89081 Ulm, Germany, uwe.knippschild@uniklinik-ulm.de

Supplement 2.6: Fatty acid content in triglyceride fraction from 1h to 21d post-injury for female obese C57BL/6J mice. N.d. = not detected.

|  | **Trigylceride fraction in muscle tissue of female obese C57BL/6J mice** | | | | | | | | | | | | | | | | | | | | | | | |
| --- | --- | --- | --- | --- | --- | --- | --- | --- | --- | --- | --- | --- | --- | --- | --- | --- | --- | --- | --- | --- | --- | --- | --- | --- |
| **Time** | **1h** | | | | **6h** | | | | **24h** | | | | **72h** | | | | **192h** | | | | **504h** | | | |
| **Treatment** | **Control** | | **Trauma** | | **Control** | | **Trauma** | | **Control** | | **Trauma** | | **Control** | | **Trauma** | | **Control** | | **Trauma** | | **Control** | | **Trauma** | |
|  | AV | sd | AV | sd | AV | sd | AV | sd | AV | sd | AV | sd | AV | sd | AV | sd | AV | sd | AV | sd | AV | sd | AV | sd |
| Myristic (14:0) | 1.19 | 0.03 | 1.09 | 0.08 | 1.30 | 0.12 | 1.21 | 0.13 | 1.27 | 0.12 | 1.18 | 0.02 | 1.05 | 0.08 | 1.16 | 0.11 | 1.17 | 0.10 | 1.12 | 0.04 | 1.16 | 0.02 | 1.16 | 0.04 |
| Myristoleic (14:1) | 0.04 | 0.01 | 0.05 | 0.01 | 0.06 | 0.02 | 0.05 | 0.01 | 0.07 | 0.03 | 0.05 | 0.00 | 0.04 | 0.00 | 0.06 | 0.00 | 0.05 | 0.00 | 0.05 | 0.01 | 0.06 | 0.01 | 0.06 | 0.01 |
| Palmitic (16:0) | 20.39 | 0.21 | 19.49 | 1.05 | 20.59 | 0.49 | 20.81 | 0.59 | 20.93 | 0.99 | 20.69 | 0.58 | 20.15 | 1.09 | 20.16 | 0.25 | 20.84 | 0.18 | 20.76 | 0.95 | 20.60 | 0.39 | 20.40 | 0.66 |
| d-7-hexadecenoic (16:1) | 0.83 | 0.05 | 0.79 | 0.01 | 0.76 | 0.05 | 0.81 | 0.03 | 0.73 | 0.04 | 0.77 | 0.03 | 0.74 | 0.03 | 0.76 | 0.03 | 0.76 | 0.03 | 0.79 | 0.04 | 0.78 | 0.04 | 0.85 | 0.08 |
| Palmitoleic (16:1) | 4.36 | 0.70 | 4.62 | 0.46 | 4.66 | 1.33 | 4.20 | 0.57 | 5.19 | 1.12 | 4.47 | 0.14 | 4.07 | 0.52 | 4.94 | 0.50 | 4.10 | 0.41 | 4.22 | 0.32 | 4.60 | 0.51 | 4.65 | 0.38 |
| Stearic (18:0) | 6.50 | 1.41 | 5.38 | 1.34 | 5.93 | 0.73 | 7.27 | 0.38 | 5.44 | 1.04 | 6.79 | 0.83 | 6.06 | 0.43 | 5.52 | 0.61 | 6.48 | 0.63 | 6.85 | 2.31 | 5.75 | 0.09 | 5.53 | 0.29 |
| Oleic (18:1) | 39.62 | 0.26 | 42.33 | 2.42 | 40.79 | 1.67 | 39.70 | 1.10 | 40.44 | 0.72 | 40.43 | 0.79 | 42.07 | 1.53 | 42.22 | 0.49 | 41.12 | 0.12 | 41.56 | 2.29 | 40.99 | 1.13 | 40.99 | 1.73 |
| Vaccenic (18:1) | 3.97 | 0.44 | 3.28 | 0.31 | 2.97 | 0.02 | 2.97 | 0.07 | 2.78 | 0.13 | 2.96 | 0.09 | 3.02 | 0.09 | 2.73 | 0.18 | 2.83 | 0.32 | 2.67 | 0.21 | 2.69 | 0.15 | 2.77 | 0.11 |
| Linoleic (18:2) | 19.91 | 0.58 | 19.87 | 0.58 | 19.63 | 1.49 | 19.67 | 0.38 | 19.76 | 0.45 | 19.35 | 0.38 | 19.60 | 0.76 | 19.29 | 0.16 | 19.38 | 0.68 | 18.87 | 1.12 | 19.91 | 0.57 | 20.28 | 1.24 |
| g-Linolenic (18:3) | 0.05 | 0.00 | 0.06 | 0.02 | 0.05 | 0.01 | 0.06 | 0.01 | 0.05 | 0.00 | 0.05 | 0.00 | 0.05 | 0.01 | 0.05 | 0.01 | 0.05 | 0.00 | 0.05 | 0.01 | 0.06 | 0.00 | 0.05 | 0.01 |
| Linolenic (18:3) | 0.92 | 0.14 | 0.83 | 0.07 | 0.86 | 0.16 | 0.95 | 0.05 | 1.09 | 0.08 | 0.98 | 0.03 | 0.88 | 0.06 | 0.90 | 0.03 | 0.96 | 0.11 | 0.87 | 0.12 | 1.04 | 0.05 | 0.92 | 0.09 |
| Stearidonic (18:4) | n.d. |  | n.d. |  | n.d. |  | n.d. |  | n.d. |  | n.d. |  | n.d. |  | n.d. |  | n.d. |  | n.d. |  | n.d. |  | n.d. |  |
| Arachidic (20:0) | 0.11 | 0.02 | 0.11 | 0.03 | 0.17 | 0.16 | 0.11 | 0.01 | 0.09 | 0.03 | 0.11 | 0.01 | 0.09 | 0.01 | 0.08 | 0.03 | 0.09 | 0.03 | 0.10 | 0.04 | 0.09 | 0.01 | 0.08 | 0.00 |
| Eicosenoic (20:1) | 0.50 | 0.04 | 0.51 | 0.08 | 0.59 | 0.20 | 0.54 | 0.07 | 0.51 | 0.03 | 0.54 | 0.02 | 0.54 | 0.05 | 0.51 | 0.10 | 0.56 | 0.11 | 0.54 | 0.05 | 0.53 | 0.07 | 0.53 | 0.03 |
| Eicosadienoic (20:2) | 0.52 | 0.03 | 0.51 | 0.06 | 0.56 | 0.08 | 0.54 | 0.03 | 0.55 | 0.04 | 0.54 | 0.02 | 0.55 | 0.01 | 0.55 | 0.03 | 0.54 | 0.05 | 0.50 | 0.02 | 0.54 | 0.01 | 0.54 | 0.02 |
| DHG-Linolenic (20:3) | 0.16 | 0.01 | 0.17 | 0.03 | 0.16 | 0.01 | 0.16 | 0.01 | 0.16 | 0.01 | 0.17 | 0.01 | 0.18 | 0.01 | 0.17 | 0.02 | 0.17 | 0.00 | 0.16 | 0.02 | 0.18 | 0.02 | 0.17 | 0.02 |
| Arachidonic (20:4) | 0.43 | 0.08 | 0.43 | 0.04 | 0.47 | 0.09 | 0.44 | 0.06 | 0.47 | 0.04 | 0.44 | 0.01 | 0.44 | 0.07 | 0.48 | 0.05 | 0.45 | 0.05 | 0.45 | 0.06 | 0.53 | 0.06 | 0.52 | 0.05 |
| Eicosatrienoic (20:3) | n.d. |  | n.d. |  | n.d. |  | n.d. |  | n.d. |  | n.d. |  | n.d. |  | n.d. |  | n.d. |  | n.d. |  | n.d. |  | n.d. |  |
| Eicosatetraenoic (20:4) | n.d. |  | n.d. |  | n.d. |  | n.d. |  | n.d. |  | n.d. |  | n.d. |  | n.d. |  | n.d. |  | n.d. |  | n.d. |  | n.d. |  |
| Eicosapentaenoic (20:5) | 0.04 | 0.01 | 0.04 | 0.00 | 0.04 | 0.01 | 0.03 | 0.01 | 0.03 | 0.00 | 0.03 | 0.00 | 0.03 | 0.01 | 0.03 | 0.01 | 0.04 | 0.00 | 0.03 | 0.00 | 0.04 | 0.00 | 0.03 | 0.01 |
| Behenic (22:0) | 0.04 | 0.01 | 0.03 | 0.02 | 0.04 | 0.00 | 0.04 | 0.01 | 0.03 | 0.00 | 0.04 | 0.00 | 0.03 | 0.00 | 0.03 | 0.00 | 0.03 | 0.01 | 0.03 | 0.01 | 0.03 | 0.00 | 0.03 | 0.00 |
| Erucic (22:1) | 0.03 | 0.01 | 0.03 | 0.01 | 0.03 | 0.00 | 0.03 | 0.01 | 0.02 | 0.00 | 0.03 | 0.00 | 0.02 | 0.00 | 0.02 | 0.01 | 0.03 | 0.01 | 0.02 | 0.01 | 0.02 | 0.00 | 0.02 | 0.00 |
| Docosapentaenoic (22:5) | 0.10 | 0.01 | 0.10 | 0.01 | 0.11 | 0.01 | 0.11 | 0.00 | 0.11 | 0.01 | 0.09 | 0.01 | 0.10 | 0.01 | 0.09 | 0.00 | 0.09 | 0.01 | 0.09 | 0.01 | 0.10 | 0.00 | 0.10 | 0.01 |
| Docosahexaenoic (22:6) | 0.22 | 0.04 | 0.22 | 0.01 | 0.24 | 0.02 | 0.24 | 0.02 | 0.25 | 0.01 | 0.23 | 0.01 | 0.23 | 0.03 | 0.24 | 0.02 | 0.23 | 0.02 | 0.24 | 0.03 | 0.28 | 0.01 | 0.27 | 0.03 |
| Lignoceric (24:0) | 0.03 | 0.01 | 0.02 | 0.01 | 0.03 | 0.01 | 0.03 | 0.01 | 0.02 | 0.01 | 0.02 | 0.01 | 0.02 | 0.01 | 0.01 | 0.01 | 0.02 | 0.00 | 0.02 | 0.01 | 0.02 | 0.01 | 0.02 | 0.00 |
| Nervonic (24:1) | 0.05 | 0.00 | 0.07 | 0.06 | 0.02 | 0.00 | 0.04 | 0.02 | 0.02 | 0.02 | 0.02 | 0.00 | 0.02 | 0.00 | 0.01 | 0.01 | 0.02 | 0.01 | 0.02 | 0.01 | 0.02 | 0.00 | 0.01 | 0.01 |
