# Supplementary material for: Comparison of Fatty Acid and Gene Profiles in Skeletal Muscle in Normal and Obese C57BL/6J Mice before and after Blunt Muscle Injury
Source: Front Physiol. 2018 Jan 30;9:19. doi: 10.3389/fphys.2018.00019 (PMC5797686; doi:10.3389/fphys.2018.00019)
Supplement: Supplement 3.1 — ID's, gene name, description and significance levels of fat metabolism microarray analysis. Red = p ≤ 0.05; Green = p ≤ 0.01. [file Supplement3.1.DOCX]

Supplementary Material

Comparison of fatty acid and gene profiles in skeletal muscle in normal and obese C57BL/6J mice before and after blunt muscle injury

Jens-Uwe Werner^1†^, Klaus Tödter^2†^, Pengfei Xu^1^, Lydia Lockhart^1^, Markus Jähnert^3^, Pascal Gottmann^3^, Annette Schürmann^3^, Ludger Scheja^2^, Martin Wabitsch^4,^*, Uwe Knippschild^1,^*

* Correspondence: Prof. Dr. Martin Wabitsch, Ulm University Hospital for Pediatrics and Adolescent Medicine, Division of Pediatric Endocrinology and Diabetes, Eythstraße 24, 89075 Ulm, Germany, martin.wabitsch@uniklinik-ulm.de and Prof. Dr. Uwe Knippschild, Ulm University Hospital, Department of General and Visceral Surgery, Albert-Einstein-Allee 23, 89081 Ulm, Germany, uwe.knippschild@uniklinik-ulm.de

Supplement 3.1: ID’s, gene name, description and significance levels of fat metabolism microarray analysis. Red = p ≤ 0.05; Green = p ≤ 0.01.

|  |  | **Trauma versus Control** | | | | | | | | | |  |
| --- | --- | --- | --- | --- | --- | --- | --- | --- | --- | --- | --- | --- |
|  |  | **Normal** | | | | | **Obese** | | | | |  |
| **UniqueID** | **Name** | **1h** | **6h** | **24h** | **3d** | **8d** | **1h** | **6h** | **24h** | **3d** | **8d** | **Description** |
| 171210 | *Acot2* | 9.447E-01 | 7.113E-01 | 7.867E-01 | 3.468E-01 | 5.394E-01 | 1.937E-01 | 6.061E-01 | 1.555E-02 | 8.928E-01 | 9.605E-01 | acyl-CoA thioesterase 2 |
| 11690 | *Alox5ap* | 6.268E-02 | 1.989E-01 | 1.907E-01 | 4.978E-03 | 1.855E-01 | 1.728E-01 | 3.770E-01 | 1.988E-01 | 2.353E-01 | 3.321E-01 | arachidonate 5-lipoxygenase activating protein |
| 11810 | *Apobec1* | 4.463E-01 | 1.379E-01 | 3.604E-01 | 2.458E-02 | 6.245E-02 | 3.047E-01 | 2.030E-01 | 1.210E-01 | 2.009E-01 | 2.038E-01 | apolipoprotein B mRNA editing enzyme, catalytic polypeptide 1 |
| 11811 | *Apobec2* | 5.740E-02 | 8.834E-01 | 1.772E-01 | 3.476E-02 | 8.850E-01 | 2.977E-01 | 2.484E-01 | 1.669E-01 | 3.413E-01 | 4.897E-01 | apolipoprotein B mRNA editing enzyme, catalytic polypeptide 2 |
| 80287 | *Apobec3* | 6.206E-01 | 3.628E-01 | 2.541E-01 | 2.235E-02 | 5.410E-02 | 4.367E-01 | 3.826E-01 | 4.207E-01 | 1.725E-01 | 3.750E-01 | apolipoprotein B mRNA editing enzyme, catalytic polypeptide 3 |
| 11816 | *Apoe* | 6.832E-01 | 9.703E-01 | 6.142E-01 | 2.107E-04 | 3.141E-02 | 4.226E-01 | 6.079E-01 | 3.972E-01 | 1.954E-01 | 6.515E-02 | apolipoprotein E |
| 54325 | *Elovl1* | 2.021E-01 | 1.711E-01 | 5.317E-01 | 1.302E-02 | 1.469E-01 | 6.440E-01 | 3.921E-01 | 2.376E-01 | 3.493E-01 | 7.470E-01 | elongation of very long chain fatty acids (FEN1/Elo2, SUR4/Elo3, yeast)-like 1 |
| 68801 | *Elovl5* | 5.274E-01 | 5.867E-01 | 5.201E-01 | 1.590E-02 | 3.791E-01 | 3.611E-01 | 8.224E-01 | 7.476E-01 | 5.809E-01 | 3.399E-01 | elongation of very long chain fatty acids (FEN1/Elo2, SUR4/Elo3, yeast)-like 2 |
| 14077 | *Fabp3* | 9.356E-01 | 8.249E-01 | 4.070E-02 | 4.700E-01 | 9.085E-01 | 6.781E-01 | 8.901E-01 | 7.666E-02 | 2.018E-01 | 6.470E-01 | elongation of very long chain fatty acids (FEN1/Elo2, SUR4/Elo3, yeast)-like 3 |
| 76267 | *Fads1* | 9.717E-01 | 4.815E-01 | 9.228E-01 | 4.549E-02 | 7.379E-01 | 9.466E-01 | 3.122E-01 | 7.456E-01 | 3.533E-01 | 8.806E-01 | elongation of very long chain fatty acids (FEN1/Elo2, SUR4/Elo3, yeast)-like 4 |
| 60527 | *Fads3* | 3.126E-01 | 8.764E-01 | 3.198E-01 | 5.360E-03 | 4.743E-02 | 4.947E-01 | 8.668E-01 | 3.166E-01 | 2.225E-01 | 8.939E-01 | ELOVL family member 5, elongation of long chain fatty acids (yeast) |
| 56356 | *Gltp* | 9.259E-01 | 5.394E-01 | 2.903E-01 | 5.778E-03 | 1.992E-02 | 3.003E-01 | 4.150E-01 | 1.809E-01 | 1.846E-01 | 3.427E-01 | ELOVL family member 6, elongation of long chain fatty acids (yeast) |
| 16819 | *Lcn2* | 1.832E-01 | 2.181E-01 | 3.525E-01 | 3.026E-02 | 3.270E-01 | 2.462E-01 | 4.289E-01 | 4.247E-01 | 1.290E-01 | 4.918E-01 | ELOVL family member 7, elongation of long chain fatty acids (yeast) |
| 16835 | *Ldlr* | 6.316E-01 | 7.290E-02 | 6.008E-01 | 2.144E-02 | 7.751E-01 | 1.170E-01 | 3.133E-01 | 1.191E-01 | 6.973E-01 | 9.534E-01 | fatty acid binding protein 1, liver |
| 16956 | *Lpl* | 5.575E-01 | 5.910E-01 | 1.160E-02 | 1.726E-01 | 3.125E-01 | 7.000E-01 | 7.571E-01 | 1.697E-01 | 1.332E-01 | 6.898E-01 | fatty acid binding protein 12 |
| 16971 | *Lrp1* | 4.425E-01 | 1.955E-01 | 8.946E-01 | 1.322E-02 | 1.190E-01 | 7.346E-01 | 9.513E-01 | 1.254E-01 | 1.728E-01 | 4.148E-01 | fatty acid binding protein 2, intestinal |
| 67620 | *Lrp2bp* | 5.649E-01 | 5.298E-01 | 7.535E-02 | 2.933E-04 | 1.073E-01 | 9.849E-01 | 2.959E-01 | 1.299E-01 | 2.831E-01 | 6.981E-02 | fatty acid binding protein 3, muscle and heart |
| 18830 | *Pltp* | 3.597E-01 | 9.606E-01 | 7.098E-01 | 1.084E-02 | 7.346E-02 | 9.973E-01 | 9.052E-01 | 4.417E-01 | 2.570E-01 | 2.721E-02 | fatty acid binding protein 4, adipocyte |
| 30049 | *Scd3* | 5.805E-01 | 9.412E-01 | 4.618E-01 | 1.028E-02 | 1.800E-02 | 5.732E-01 | 2.025E-01 | 1.811E-01 | 1.748E-01 | 9.911E-01 | fatty acid binding protein 5, epidermal |
| 20787 | *Srebf1* | 7.391E-01 | 7.842E-01 | 8.018E-01 | 3.546E-02 | 1.379E-01 | 8.309E-01 | 4.709E-01 | 9.780E-01 | 7.890E-01 | 9.499E-02 | fatty acid binding protein 6, ileal (gastrotropin) |
| 20788 | *Srebf2* | 7.814E-01 | 3.854E-01 | 9.599E-02 | 1.867E-02 | 2.415E-01 | 4.256E-01 | 3.094E-01 | 3.168E-02 | 5.817E-01 | 3.986E-01 | fatty acid binding protein 7, brain |
