# Supplementary material for: Comparison of Fatty Acid and Gene Profiles in Skeletal Muscle in Normal and Obese C57BL/6J Mice before and after Blunt Muscle Injury
Source: Front Physiol. 2018 Jan 30;9:19. doi: 10.3389/fphys.2018.00019 (PMC5797686; doi:10.3389/fphys.2018.00019)
Supplement: Supplement 3.2 — ID's, gene name, description and significance levels of Notch, Insulin, Sonic hedgehog and Apoptosis microarray analysis. Red = p ≤ 0.05; Green = p ≤ 0.01. [file Supplement3.2.DOCX]

Supplementary Material

Comparison of fatty acid and gene profiles in skeletal muscle in normal and obese C57BL/6J mice before and after blunt muscle injury

Jens-Uwe Werner^1†^, Klaus Tödter^2†^, Pengfei Xu^1^, Lydia Lockhart^1^, Markus Jähnert^3^, Pascal Gottmann^3^, Annette Schürmann^3^, Ludger Scheja^2^, Martin Wabitsch^4,^*, Uwe Knippschild^1,^*

* Correspondence: Prof. Dr. Martin Wabitsch, Ulm University Hospital for Pediatrics and Adolescent Medicine, Division of Pediatric Endocrinology and Diabetes, Eythstraße 24, 89075 Ulm, Germany, martin.wabitsch@uniklinik-ulm.de and Prof. Dr. Uwe Knippschild, Ulm University Hospital, Department of General and Visceral Surgery, Albert-Einstein-Allee 23, 89081 Ulm, Germany, uwe.knippschild@uniklinik-ulm.de

Supplement 3.2: ID’s, gene name, description and significance levels of Notch, Insulin, Sonic hedgehog and Apoptosis microarray analysis. Red = p ≤ 0.05; Green = p ≤ 0.01.

|  |  | **Trauma versus Control** | | | | | | | | | |  |
| --- | --- | --- | --- | --- | --- | --- | --- | --- | --- | --- | --- | --- |
|  |  | **Normal** | | | | | **Obese** | | | | |  |
| **UniqueID** | **Gene** | **1h** | **6h** | **24h** | **3d** | **8d** | **1h** | **6h** | **24h** | **3d** | **8d** | **Description** |
| **Notch** | |  |  |  |  |  |  |  |  |  |  |  |
| 11491 | *Adam17* | 1.47E-01 | 3.91E-01 | 3.03E-02 | 2.38E-04 | 4.92E-01 | 4.14E-01 | 4.49E-01 | 8.66E-02 | 2.35E-01 | 2.18E-01 | a disintegrin and metallopeptidase domain 17 |
| 11492 | *Adam19* | 6.32E-02 | 5.69E-01 | 3.81E-01 | 9.40E-03 | 1.93E-02 | 1.39E-01 | 7.64E-01 | 4.64E-01 | 2.20E-01 | 9.61E-02 | a disintegrin and metallopeptidase domain 19 (meltrin beta) |
| 16848 | *Lfng* | 7.05E-01 | 6.45E-01 | 1.23E-01 | 3.21E-02 | 8.48E-02 | 1.25E-01 | 6.23E-01 | 1.50E-02 | 1.60E-01 | 3.81E-01 | LFNG O-fucosylpeptide 3-beta-N-acetylglucosaminyltransferase |
| 18129 | *Notch2* | 2.33E-01 | 1.26E-01 | 9.63E-01 | 5.69E-03 | 7.61E-02 | 5.86E-01 | 6.28E-01 | 7.94E-02 | 2.32E-01 | 1.75E-01 | notch 2 |
| **Insulin** | |  |  |  |  |  |  |  |  |  |  |  |
| 11651 | *Akt1* | 6.73E-01 | 3.38E-01 | 1.18E-01 | 2.80E-02 | 1.18E-01 | 8.11E-01 | 3.93E-01 | 6.76E-02 | 6.27E-01 | 9.86E-01 | thymoma viral proto-oncogene 1 |
| 54601 | *Foxo4* | 7.59E-01 | 3.78E-01 | 1.09E-01 | 1.99E-02 | 2.39E-03 | 2.19E-01 | 4.65E-01 | 3.93E-01 | 9.33E-01 | 8.26E-01 | forkhead box O4 |
| 14360 | *Fyn* | 1.20E-01 | 7.30E-01 | 2.40E-01 | 4.90E-03 | 1.31E-04 | 6.37E-01 | 4.30E-01 | 7.51E-01 | 7.18E-01 | 1.29E-01 | Fyn proto-oncogene |
| 16331 | *Inpp5d* | 6.77E-02 | 2.95E-02 | 1.73E-01 | 1.92E-02 | 1.05E-01 | 4.06E-01 | 2.75E-01 | 1.04E-01 | 1.96E-01 | 5.93E-01 | inositol polyphosphate-5-phosphatase D |
| 228026 | *Pdk1* | 7.68E-01 | 5.15E-01 | 1.07E-02 | 6.76E-03 | 1.30E-02 | 3.85E-01 | 3.98E-01 | 3.62E-02 | 3.03E-01 | 5.23E-01 | pyruvate dehydrogenase kinase, isoenzyme 1 |
| 18604 | *Pdk2* | 4.53E-02 | 2.53E-01 | 5.29E-02 | 2.95E-05 | 4.27E-03 | 8.39E-01 | 1.34E-01 | 4.21E-02 | 1.17E-01 | 2.48E-01 | pyruvate dehydrogenase kinase, isoenzyme 2 |
| 236900 | *Pdk3* | 5.36E-01 | 1.22E-01 | 1.16E-01 | 7.93E-03 | 1.71E-01 | 4.73E-01 | 5.00E-01 | 7.96E-01 | 8.57E-01 | 8.77E-01 | pyruvate dehydrogenase kinase, isoenzyme 3 |
| 27273 | *Pdk4* | 7.56E-01 | 6.72E-01 | 6.94E-01 | 8.73E-01 | 4.57E-01 | 2.40E-01 | 4.07E-01 | 1.81E-02 | 7.07E-01 | 9.08E-01 | pyruvate dehydrogenase kinase, isoenzyme 4 |
| 19246 | *Ptpn1* | 2.09E-01 | 5.27E-02 | 6.86E-02 | 4.69E-03 | 2.69E-01 | 6.23E-01 | 4.94E-01 | 1.58E-01 | 5.81E-02 | 7.20E-01 | protein tyrosine phosphatase, non-receptor type 1 |
| 110157 | *Raf1* | 1.99E-01 | 3.38E-01 | 1.42E-01 | 6.40E-04 | 5.80E-02 | 2.31E-01 | 2.84E-01 | 6.13E-01 | 3.98E-01 | 3.11E-01 | v-raf-leukemia viral oncogene 1 |
| 170755 | *Sgk3* | 2.82E-01 | 3.40E-01 | 4.88E-02 | 1.98E-04 | 9.92E-01 | 2.06E-03 | 4.42E-01 | 3.43E-01 | 4.40E-01 | 3.15E-01 | serum/glucocorticoid regulated kinase 3 |
| **Sonic hedgehog** | |  |  |  |  |  |  |  |  |  |  |  |
| 83396 | *Glis2* | 2.86E-01 | 1.42E-01 | 1.58E-02 | 3.86E-02 | 1.22E-02 | 6.30E-01 | 6.61E-01 | 8.98E-01 | 4.60E-01 | 9.70E-01 | GLIS family zinc finger 2 |
| 17869 | *Myc* | 1.48E-02 | 1.94E-01 | 6.68E-02 | 5.38E-02 | 3.38E-01 | 4.57E-02 | 2.61E-01 | 1.36E-01 | 9.08E-01 | 1.88E-01 | myelocytomatosis oncogene |
| **Apoptosis** | |  |  |  |  |  |  |  |  |  |  |  |
| 12018 | *Bak1* | 1.05E-01 | 9.24E-01 | 1.35E-02 | 1.07E-03 | 1.79E-02 | 2.35E-01 | 1.75E-01 | 1.93E-01 | 2.05E-01 | 5.41E-01 | BCL2-antagonist/killer 1 |
| 12028 | *Bax* | 5.05E-01 | 2.29E-01 | 3.32E-01 | 6.91E-03 | 9.96E-02 | 8.03E-01 | 8.86E-01 | 1.59E-01 | 6.83E-01 | 8.56E-01 | BCL2-associated X protein |
| 12122 | *Bid* | 6.03E-01 | 1.62E-01 | 6.77E-02 | 1.80E-02 | 1.35E-01 | 6.61E-01 | 9.53E-01 | 4.39E-01 | 2.91E-01 | 6.80E-01 | BH3 interacting domain death agonist |
| 12334 | *Capn2* | 6.86E-02 | 1.74E-02 | 3.24E-01 | 3.02E-03 | 1.05E-01 | 4.84E-01 | 4.33E-01 | 8.11E-01 | 1.87E-01 | 5.33E-02 | calpain 2 |
| 12338 | *Capn6* | 4.95E-03 | 2.49E-01 | 3.80E-02 | 1.47E-02 | 8.99E-02 | 9.82E-01 | 1.66E-01 | 2.76E-01 | 3.31E-01 | 3.88E-01 | calpain 6 |
| 12339 | *Capn7* | 8.75E-01 | 8.31E-01 | 1.60E-01 | 1.21E-02 | 2.83E-03 | 2.89E-01 | 8.58E-01 | 3.01E-01 | 8.00E-01 | 9.69E-01 | calpain 7 |
| 12367 | *Casp3* | 9.87E-01 | 5.26E-01 | 1.73E-01 | 5.38E-03 | 4.96E-02 | 8.06E-01 | 4.41E-01 | 1.16E-01 | 1.93E-01 | 7.50E-01 | caspase 3 |
| 12368 | *Casp6* | 8.78E-01 | 7.72E-02 | 2.62E-01 | 1.06E-02 | 7.10E-02 | 2.74E-01 | 2.57E-01 | 9.04E-01 | 3.80E-01 | 9.88E-02 | caspase 6 |
| 12370 | *Casp8* | 3.67E-01 | 2.41E-01 | 8.56E-02 | 1.85E-02 | 3.21E-01 | 7.33E-01 | 3.57E-01 | 2.06E-01 | 2.36E-01 | 3.70E-01 | caspase 8 |
| 12534 | *Cdk1* | 1.49E-01 | 1.67E-01 | 2.72E-01 | 2.36E-02 | 3.14E-01 | 9.79E-01 | 1.64E-01 | 8.95E-01 | 1.72E-01 | 8.82E-01 | cyclin-dependent kinase 1 |
| 67454 | *Ikbip* | 7.86E-01 | 9.08E-01 | 1.46E-01 | 4.84E-02 | 4.48E-01 | 9.25E-01 | 9.73E-01 | 6.83E-02 | 2.30E-01 | 6.44E-01 | IKBKB interacting protein |
| 16477 | *Junb* | 7.97E-02 | 2.78E-01 | 8.00E-01 | 4.98E-03 | 4.32E-01 | 9.72E-02 | 1.40E-01 | 4.34E-03 | 3.25E-01 | 8.62E-01 | jun B proto-oncogene |
| 16478 | *Jund* | 4.00E-02 | 1.89E-01 | 2.84E-01 | 2.85E-01 | 1.87E-01 | 4.86E-02 | 5.14E-01 | 4.20E-01 | 9.38E-01 | 5.16E-01 | jun D proto-oncogene |
| 18033 | *Nfkb1* | 1.03E-01 | 1.51E-01 | 8.20E-01 | 1.36E-03 | 4.79E-01 | 3.60E-01 | 1.87E-01 | 5.62E-01 | 4.26E-01 | 8.10E-01 | nuclear factor of kappa light polypeptide gene enhancer in B cells 1, p105 |
| 243910 | *Nfkbid* | 1.78E-01 | 7.83E-01 | 9.63E-01 | 5.51E-01 | 3.64E-01 | 1.17E-02 | 5.76E-01 | 5.36E-01 | 4.58E-01 | 6.90E-01 | nuclear factor of kappa light polypeptide gene enhancer in B cells inhibitor, delta |
| 18037 | *Nfkbie* | 6.11E-02 | 8.12E-01 | 2.23E-01 | 1.43E-02 | 2.36E-01 | 4.34E-02 | 7.16E-01 | 5.00E-01 | 1.78E-01 | 7.14E-02 | nuclear factor of kappa light polypeptide gene enhancer in B cells inhibitor, epsilon |
| 80859 | *Nfkbiz* | 1.19E-01 | 2.81E-01 | 8.10E-01 | 3.24E-02 | 8.08E-01 | 3.97E-02 | 3.08E-01 | 5.15E-01 | 6.96E-02 | 2.47E-01 | nuclear factor of kappa light polypeptide gene enhancer in B cells inhibitor, zeta |
| 19766 | *Ripk1* | 6.72E-03 | 4.45E-03 | 8.65E-01 | 6.31E-03 | 3.82E-02 | 5.93E-02 | 9.00E-02 | 2.26E-01 | 2.47E-01 | 3.23E-01 | receptor (TNFRSF)-interacting serine-threonine kinase 1 |
| 19877 | *Rock1* | 1.20E-01 | 2.19E-01 | 2.68E-01 | 1.87E-02 | 1.96E-01 | 8.57E-01 | 3.29E-01 | 9.84E-01 | 8.36E-01 | 9.95E-02 | Rho-associated coiled-coil containing protein kinase 1 |
| 22059 | *Trp53* | 7.26E-01 | 8.86E-01 | 5.18E-03 | 1.26E-01 | 2.98E-02 | 6.87E-01 | 3.63E-01 | 4.01E-01 | 4.54E-01 | 2.65E-01 | transformation related protein 53 |
| 60599 | *Trp53inp1* | 5.38E-01 | 6.65E-01 | 2.61E-01 | 9.75E-01 | 2.99E-01 | 9.68E-01 | 4.94E-01 | 4.03E-02 | 5.17E-01 | 6.25E-01 | transformation related protein 53 inducible nuclear protein 1 |
| **Shared genes** | |  |  |  |  |  |  |  |  |  |  |  |
| 67605 | *Akt1s1* | 1.53E-01 | 9.45E-01 | 2.39E-01 | 3.13E-03 | 1.33E-02 | 6.58E-01 | 4.10E-01 | 8.52E-02 | 8.06E-01 | 1.75E-01 | AKT1 substrate 1 (proline-rich) |
| 23797 | *Akt3* | 4.30E-02 | 7.93E-01 | 4.83E-02 | 4.65E-02 | 6.93E-02 | 9.73E-01 | 5.02E-01 | 7.26E-02 | 7.96E-01 | 3.20E-01 | thymoma viral proto-oncogene 3 |
| 26417 | *Mapk3* | 4.51E-01 | 2.60E-01 | 3.47E-01 | 2.22E-02 | 3.15E-01 | 3.03E-02 | 6.06E-01 | 9.01E-01 | 3.87E-01 | 7.28E-01 | mitogen-activated protein kinase 3 |
| 18704 | *Pik3c2a* | 2.86E-01 | 4.98E-01 | 8.16E-01 | 3.17E-02 | 2.50E-01 | 5.29E-01 | 2.31E-01 | 7.46E-01 | 3.36E-01 | 1.77E-01 | phosphatidylinositol 3-kinase, C2 domain containing, alpha polypeptide |
| 30955 | *Pik3cg* | 3.82E-02 | 1.62E-01 | 7.26E-02 | 8.77E-03 | 2.28E-01 | 5.34E-02 | 2.17E-01 | 2.16E-01 | 1.91E-01 | 5.13E-01 | phosphoinositide-3-kinase, catalytic, gamma polypeptide |
| 216505 | *Pik3ip1* | 6.48E-01 | 3.17E-01 | 1.88E-03 | 2.10E-02 | 6.33E-03 | 4.66E-01 | 2.26E-01 | 1.95E-01 | 9.51E-01 | 9.12E-01 | phosphoinositide-3-kinase interacting protein 1 |
| 320207 | *Pik3r5* | 7.74E-02 | 3.90E-01 | 4.73E-01 | 3.08E-02 | 9.41E-02 | 5.80E-02 | 2.70E-01 | 9.79E-02 | 2.05E-01 | 3.44E-01 | phosphoinositide-3-kinase, regulatory subunit 5, p101 |
| 18750 | *Prkca* | 5.26E-01 | 4.72E-01 | 6.30E-01 | 1.14E-02 | 5.74E-03 | 7.70E-01 | 5.24E-01 | 3.30E-02 | 7.26E-01 | 7.28E-01 | protein kinase C, alpha |
| 18751 | *Prkcb* | 3.27E-01 | 7.31E-01 | 3.14E-01 | 9.23E-04 | 7.17E-03 | 3.56E-01 | 2.03E-01 | 3.77E-01 | 1.06E-01 | 5.96E-01 | protein kinase C, beta |
| 18753 | *Prkcd* | 2.57E-01 | 7.23E-03 | 3.62E-01 | 2.72E-02 | 3.02E-02 | 8.54E-01 | 2.48E-01 | 3.23E-01 | 1.76E-01 | 3.52E-01 | protein kinase C, delta |
| 109042 | *Prkcdbp* | 1.12E-01 | 3.54E-01 | 7.50E-02 | 1.82E-02 | 3.07E-01 | 3.00E-01 | 8.73E-01 | 7.11E-02 | 3.06E-01 | 9.94E-01 | protein kinase C, delta binding protein |
| 18755 | *Prkch* | 9.09E-01 | 1.16E-01 | 4.92E-01 | 4.29E-02 | 7.27E-01 | 3.60E-01 | 4.19E-01 | 7.58E-01 | 3.73E-01 | 2.80E-01 | protein kinase C, eta |
| 18759 | *Prkci* | 4.19E-01 | 5.29E-01 | 3.95E-01 | 1.01E-02 | 1.68E-01 | 8.59E-01 | 2.32E-01 | 3.11E-01 | 3.59E-01 | 4.34E-01 | protein kinase C, iota |
| 18761 | *Prkcq* | 3.88E-01 | 5.40E-01 | 2.47E-01 | 1.13E-02 | 5.62E-01 | 1.57E-01 | 5.88E-01 | 1.47E-01 | 2.35E-01 | 3.16E-01 | protein kinase C, theta |
| 19089 | *Prkcsh* | 8.92E-01 | 5.71E-01 | 5.67E-01 | 7.94E-04 | 8.22E-01 | 3.32E-01 | 8.20E-01 | 4.09E-01 | 9.23E-01 | 5.82E-01 | protein kinase C substrate 80K-H |
| 105787 | *Prkaa1* | 5.62E-01 | 6.89E-01 | 5.10E-02 | 1.67E-02 | 5.27E-02 | 7.46E-01 | 6.70E-01 | 2.59E-01 | 9.73E-01 | 5.21E-01 | protein kinase, AMP-activated, alpha 1 catalytic subunit |
| 108079 | *Prkaa2* | 7.56E-01 | 6.39E-01 | 1.72E-01 | 1.63E-04 | 8.83E-03 | 1.70E-01 | 3.30E-01 | 9.03E-01 | 3.80E-01 | 2.90E-01 | protein kinase, AMP-activated, alpha 2 catalytic subunit |
| 108097 | *Prkab2* | 4.67E-01 | 4.07E-01 | 8.76E-01 | 2.14E-02 | 3.16E-02 | 1.13E-01 | 5.13E-01 | 7.65E-01 | 5.68E-02 | 2.10E-01 | protein kinase, AMP-activated, beta 2 non-catalytic subunit |
| 19082 | *Prkag1* | 5.87E-02 | 1.58E-01 | 2.33E-01 | 1.05E-03 | 1.04E-01 | 9.62E-01 | 1.75E-01 | 9.64E-01 | 3.27E-02 | 3.30E-01 | protein kinase, AMP-activated, gamma 1 non-catalytic subunit |
| 241113 | *Prkag3* | 3.45E-01 | 2.16E-01 | 9.93E-02 | 1.47E-02 | 5.87E-01 | 2.79E-01 | 3.01E-01 | 9.01E-02 | 3.61E-01 | 9.41E-01 | protein kinase, AMP-activated, gamma 3 non-catatlytic subunit |
| 19087 | *Prkar2a* | 1.19E-01 | 2.54E-01 | 6.45E-01 | 1.38E-02 | 3.31E-02 | 2.72E-01 | 4.30E-01 | 2.93E-01 | 7.62E-01 | 3.93E-01 | protein kinase, cAMP dependent regulatory, type II alpha |
